# Recent Advance in Solution‐Processed Organic Interlayers for High‐Performance Planar Perovskite Solar Cells

**DOI:** 10.1002/advs.201800159

**Published:** 2018-05-08

**Authors:** Wenxiao Zhang, Ying‐Chiao Wang, Xiaodong Li, Changjian Song, Li Wan, Khurram Usman, Junfeng Fang

**Affiliations:** ^1^ Key Laboratory of Graphene Technologies and Applications of Zhejiang Province Ningbo Institute of Materials Technology and Engineering Chinese Academy of Sciences Ningbo 315201 China; ^2^ University of Chinese Academy of Sciences Beijing 100049 China

**Keywords:** organic interlayers, perovskite solar cells, planar, polymers, small molecules

## Abstract

Planar heterojunction perovskite solar cells (PSCs) provide great potential for fabricating high‐efficiency, low‐cost, large‐area, and flexible photovoltaic devices. In planar PSCs, a perovskite absorber is sandwiched between hole and electron transport materials. The charge‐transporting interlayers play an important role in enhancing charge extraction, transport, and collection. Organic interlayers including small molecules and polymers offer great advantages for their tunable chemical/electronic structures and low‐temperature solution processibility. Here, recent progress of organic interlayers in planar heterojunction PSCs is discussed, and the effect of chemical structures on device performance is also illuminated. Finally, the main challenges in developing planar heterojunction PSCs based on organic interlayers are identified, and strategies for enhancing the device performance are also proposed.

## 1 Introduction

Organometal halide perovskite solar cells (PSCs) have pushed forward the photovoltaics progress since first reported by Miyasaka and co‐workers in 2009 and their power conversion efficiency (PCE) has exceeded 22% in only a few years.^1^, ^2^, ^3^, ^4^, ^5^ The remarkable performance of perovskites in solar cells is attributed to their impressive electrical and optical properties, such as the high absorption extinction coefficient in the visible‐near infrared wavelength region,^6^, ^7^ direct bandgap, high dielectric constant,^8^ long carrier diffusion length,^9^, ^10^ and excellent high charge carrier mobility.^11^, ^12^ PSCs are classified into two types of device architechtures: regular (n–i–p) and inverted (p–i–n) structures depending on whether electrons or holes are collected at the bottom conducting substrate. Much progress has been made through improving perovskite film quality, by using the strategies including vapor‐phase deposition techniques,^13^ vapor‐assisted solution process,^14^ solvent chemical engineering,^15^ and annealing treatment.^16^, ^17^ In addition, interlayers also play important role in advancing PSCs development. Most high efficient PSCs have been dominated by the device architecture with mesoporous metal oxide scaffolds (e.g., TiO_2_ and Al_2_O_3_) under perovskite layer.^18^, ^19^, ^20^, ^21^ However, the preparation of mesoporous metal oxides typically requires high sintering temperature over 450 °C to create the conductive phase. In addition, the homogeneous porous frameworks are difficult to control.^22^, ^23^ These factors limited their application in flexible and large‐scale industrialized PSCs. To achieve fully low‐temperature solution‐processed PSCs, two schemes were designed. One is to replace mesoporous metal oxides with low temperature processed dense metal oxides or organic interlayers, such as TiO_2_ nanorods,^24^ anatase TiO_2_ small nanoparticles,^25^ ZnO,^26^ SnO_2_,^27^ and fullerene‐based materials^28^ in regular planar cells. The other strategy is to employ an inverted PSC based on fully organic interlayers.^29^, ^30^ Among these alternative interfacial materials, organic interlayers exhibit unique characteristics including flexible, low‐temperature solution processibility, and good electrical and structural property tunability. Therefore, they show immense potential to propel PSC fields to future advancement.

**Figure 1:**
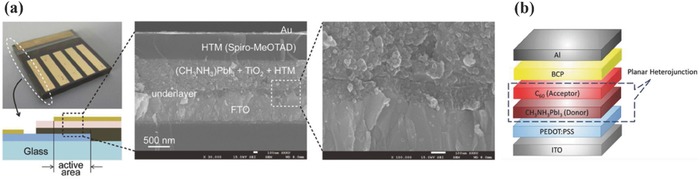
a) First solid‐state PSC device and its cross‐sectional structure with Spiro‐OMeTAD HTM. Reproduced with permission.^33^ Copyright 2012, Royal Society of Chemistry. b) Schematic device structure of the first p–i–n PSC with all organic interlayers. Reproduced with permission.^29^ Copyright 2013, John Wiley and Sons.

In virtue to their designable energy band structure, controllable interfacial wettability, tunable carrier mobility, and permanent interface dipole action,^31^, ^32^ the solution‐processed organic interlayers play various roles in both regular and inverted PSCs. The first full‐solid‐state PSC device showed significant improvement of device performance and chemical stability by using an organic hole transport materials (HTM) 2,2′,7,7′‐tetrakis(N,N‐dipmethoxyphenylamine)‐9,9′‐spirobifluorene (Spiro‐OMeTAD) to replace high corrosive liquid electrolyte, as shown in **Figure** **1**a.^33^ Later, Snaith and co‐workers used a solution‐processed fullerene compact layer forming an n‐type selective contact at cathode, entirely removing TiO_2_ in the regular planar devices.^28^ Many reviews have summarized the roles and function mechanism of fullerene based materials.^34^, ^35^, ^36^ The passivation effect of them and efficient electron transfer with perovskite active layer could reduce the hysteresis as well as enhance device performance. Chen and co‐workers used fully organic interlayers to implement the operation of inverted PSC devices, as shown in Figure 1b.^29^ Choosing HTMs with proper surface energy could enhance crystallization kinetics and film quality of perovskite, which results in improved performance of inverted devices.^37^, ^38^ Apart from benefits on the PCE, organic interlayers could also improve the device stability. Using hydrophobic, cross‐linked, and doped materials at perovskite/electrode interface relieves perovskite degradation in ambient conditions, or the interfacial interaction between perovskite and interlayers or metal electrode.^39^ Lately, a highly stable planar PSC was fabricated by appropriately modifying each interface and contact layer. And 94% of its peak efficiency was retained after 1000 h of continuous, unencapsulated ambient operation.^40^

In this review, we summarize the recent progresses of organic interlayers in planar PSCs and highlight the effect of their chemical structures on the device performance. This article consists of two main sections: HTMs and electron transport materials (ETMs), both including small molecules and polymers. In each section, we demonstrate different status and trends of organic interlayers in regular and inverted architectures, and illustrate various functions of them. Moreover, their virtual functions and action mechanisms on developing high efficiency, hysteresis‐free, long‐term stable, large‐area, and flexible PSCs have also been discussed. In addition to understanding the current development of organic interlayers in perovskite photovoltaic fields, we also hope to provide perspectives for rational molecular design to further improve the performance of PSCs.

## 2 Hole Transport Materials

### 2.1 Small Molecules

#### 2.1.1 n–i–p Regular Structure

**Figure 2:**
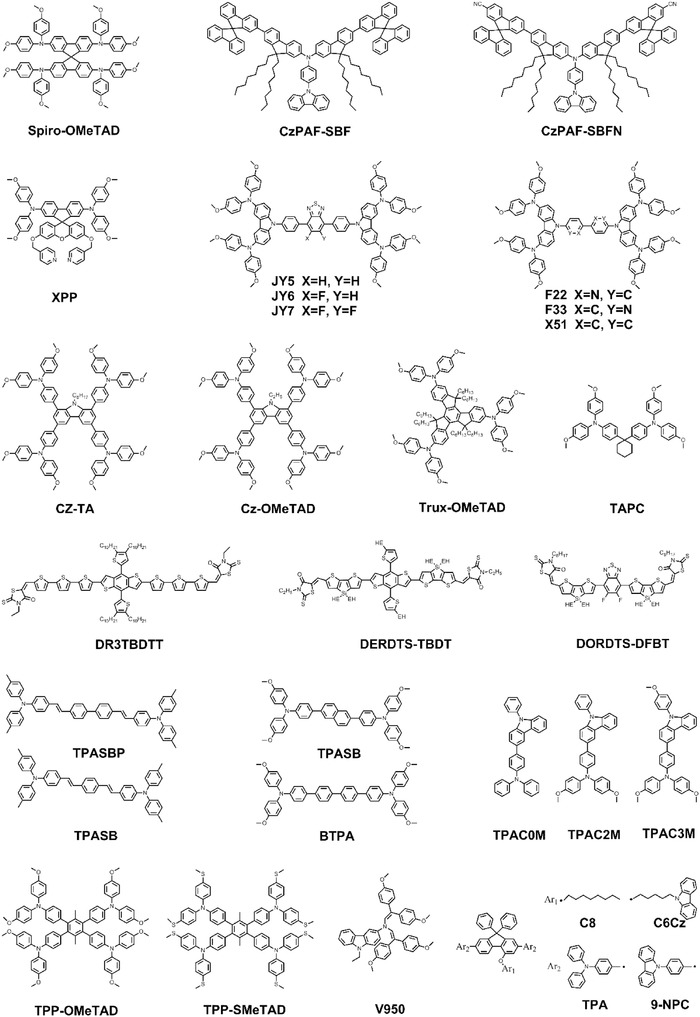
Molecular structures of small molecular HTMs.

Spiro‐OMeTAD (**Figure** **2**) has been one of the most commonly used and successful HTMs in solid state dye sensitized solar cells.^41^ Park and co‐workers used this solid state counterpart to replace liquid electrolyte in solid‐state mesoscopic heterojunction PSCs, which not only increased the device stability but remarkably increased the PCE from 3.9% to 9.7%.^1^, ^18^ The pristine form of Spiro‐OMeTAD suffers from low hole mobility on the order of 1 × 10^−4^ cm^2^ V^−1^ s^−1^ and poor conductivity on the order of 1 × 10^−5^ S cm^−1^ attributing to its inherent triangular pyramid configuration with large intermolecular distances.^42^ This leads to high series resistance (*R*_s_), low recombination resistance (*R*_rec_), and unbalanced carriers transport between anode and cathode, which causes inferior photovoltaic performance. To achieve higher conductivity and better device performance, proper doping of Spiro‐OMeTAD is required. The commonly used additives are lithium bis(trifluoromethylsulfonyl)imide salt (Li‐TFSI), tert‐butylpyridine (TBP), and tris(2‐(1H‐pyrazol‐1‐yl)‐4‐tertbutylpyridine)–cobalt(III) tris(bis(trifluoromethylsulfonyl)imide) (FK209).^13^, ^26^, ^27^, ^43^, ^44^ Li‐TFSI is an efficient and stable p‐type dopant in the presence of oxygen and leads to the formation of oxidized Spiro‐OMeTAD. The low ionization potential of Spiro‐OMeTAD and the high electron affinity of the molecular oxygen lead to an effective electron transfer to the oxygen molecule through light or thermal excitation. The formed Spiro‐OMeTAD radical cation (Spiro‐OMeTAD^+^) is weakly bound by the highly delocalized charge on the TFSI^−^, which results in an effective generation of mobile holes on the organic matrix.^45^ TBP could interact with perovskite, which causes HTM/perovskite interface to become more selective for holes.^46^ Moreover, the addition of TBP to HTM also improves the uniformity of HTM and avoids accumulation of Li salt.^47^ FK209 could lower Fermi level of Spiro‐OMeTAD HTM due to the increased hole concentration and reduce charge recombination, which increases the *V*_oc_ of devices.^48^ Snaith and co‐workers demonstrated a planar device structure with vapor‐deposited perovskite absorbing layer sandwiched between compact TiO_2_ ETM and Spiro‐OMeTAD HTM (doped with Li‐TFSI and TBP additives) yielding a PCE over 15%.^13^ Later, other planar PSCs with different ETMs, such as yttrium‐doped TiO_2_ (Y‐TiO_2_), ZnO nanoparticles, and nanocrystalline SnO_2_, and both with cobalt and lithium codoped Spiro‐OMeTAD as HTM also achieved high efficiency near to 20%.^27^, ^44^, ^49^ These studies prove that the doped Spiro‐OMeTAD can be an excellent HTM in planar structure devices with matchable energy level, good conductivity, and nonreactivity to perovskite active layer. However, the doped Spiro‐OMeTAD with Li‐TFSI require oxygen doping process and the concentration of oxidized Spiro‐OMeTAD in the HTMs is uncontrollable.^45^, ^50^ This oxidation reaction process reduces device stability and reproducibility substantially, as well as complicates device fabrication procedure. With regard to these issues, some inorganic semiconductor materials with good chemical stability and high hole mobility show potential application as HTMs in PSCs.^51^, ^52^ To combine the advantages of matchable energy levels of Spiro‐OMeTAD and good stability of inorganic materials via a solution process, Li et al. incorporated copper salts (cuprous thiocyanate (CuSCN) or cuprous iodide (CuI)) into Spiro‐OMeTAD to realize a p‐type doping with efficient charge transfer complex.^53^ The composite film showed improved film conductivity and hole mobility, which increased PCE of derived devices largely from 14.82% to 18.02%. In addition, the inorganic copper salts could also inhibit aggregation and crystallization of Spiro‐OMeTAD films with reduced pinholes and voids, which avoided the moisture infiltration to some extent and slowed down the perovskite decomposition significantly.

**Figure 3:**
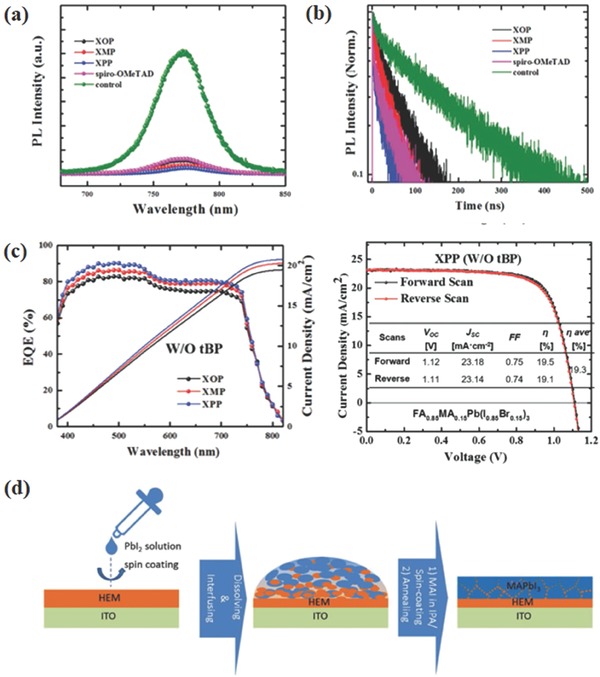
a) Steady‐state photoluminescence (PL) decay and b) the time‐resolved PL measurements without and with different HTMs. c) *J*–*V* characteristics of the champion PSC devices with different HTMs without additives and *J*–*V* characteristics of the mix‐ion PSC devices employing XPP as HTM without TBP as additive. Reproduced with permission.^57^ Copyright 2017, John Wiley and Sons. d) A schematic diagram of the deposition method and the schematic architecture of perovskite–HTM heterojunction device. Reproduced with permission.^73^ Copyright 2017, John Wiley and Sons.

All photovoltaic parameters of perovskite solar cells based small molecular HTMs with dopants are listed in **Table** **1**. In pursuit of cost effective and dopant free HTMs, various groups have designed alternative small molecule based materials by proper molecular design. In regular PSCs, Reddy and co‐workers designed and synthesized two small molecular HTMs, namely, 7‐(9,9′‐spirobifluorene‐2‐yl)‐N‐(7‐(9,9′‐spirobifluorene‐2‐yl)‐9,9‐dioctyl‐9H‐fluoren‐2‐yl)‐N‐(4‐(9H‐carbazol‐9‐yl)phenyl)‐9,9‐dioctyl‐9H‐fluoren‐2‐amine (CzPAF‐SBF) and 7‐(7′‐carbonitrile‐9,9′‐spirobifluorene‐2‐yl)‐N‐(7‐(7′‐carbonitrile‐9,9′‐spirobifluorene‐2‐yl)‐9,9‐dioctyl‐9H‐fluoren‐2‐yl)‐N‐(4‐(9H‐carbazol‐9‐yl)phenyl)‐9,9‐dioctyl‐9H‐fluoren‐2‐amine (CzPAF‐SBFN) (Figure 2), possessing triphenylamine, dioctylfluorene, and spirobifluorene as the core, spacer, and end‐caps, respectively.^54^ The two molecules showed suitable energy levels, high hole mobility with Li salt doping, and good solubility, which enable both of them to be excellent HTMs in PSCs, especially CzPAF‐SBF. The devices based on CzPAF‐SBF HTM delivered a high PCE of 17.21% and an outstanding device stability. Zhu et al. synthesized two non‐Spiro‐type small molecules, named JY6 and JY7 (Figure 2), with fluorinated benzothiadiazole (BT) electron donating core structure via simple synthetic process.^55^ JY6 with monofluorinated BT showed suitable energy levels, excellent thermal stability, and high hole mobility doped with Li‐TFSI and TBP. Thus, JY6 HTM‐based PSCs reached a high PCE of 18.54% with a remarkable fill factor (FF) value of 81%. However, the JY7‐based devices showed inferior performance mostly due to their poor film‐forming properties. The same group also used electron‐deficient bipyridine to tune the energy levels of HTMs despite the electron donating units such as BT unit that are preferable when devising HTMs especially with twisted structure.^23^, ^56^ The two HTMs with 2,2′‐ and 3,3′‐bipyridine as the core structure named F22 and F33 (Figure 2) showed matched highest occupied molecule orbital (HOMO) levels and the derived devices achieved high open‐circuit voltage (*V*_oc_: 1.05 V for F22 and 1.11 V for F33) and high FF (79% for F22 and F33). The two works showed that the fluorinated BT core‐based and bipyridine core‐based HTM were competitive materials for constructing HTMs. However, new HTMs mentioned above all require Li‐TFSI and TBP dopants to enhance hole mobility. To get rid of TBP dopant, Jin and co‐workers synthesized a series of spiro[fluorene‐9,9′‐xanthene] (SFX)‐based materials with covalently linked pyridine groups.^57^ Among these materials, N_2_,N_2_′,N_7_,N_7_′‐tetrakis(4‐methoxyphenyl)‐3′,6′‐bis(pyridin‐4‐ylmethoxy) spiro[fluorene‐9,9′‐xanthene]‐2,7‐diamine (XPP, Figure 2) with two parasubstituted pyridines on the SFX core unit showed much better hole extraction and transfer capability at perovskite/HTM interface, proved by steady‐state photoluminescence (PL) and time‐resolved PL measurements in **Figure** **3**a,b. Thus, the device performance with SFX HTM reached 17.2% based MA_3_PbI_3_ active layer and 19.5% based FA_0.85_MA_0.15_Pb[I_0.85_Br_0.15_]_3_ active layer, which outperformed that of Spiro‐OMeTAD‐based devices (Figure 3c). Most importantly, the XPP‐based PSCs also exhibited much better long‐term stability in the absence of low‐boiling point TBP as additive.

**Table 1:** Photovoltaic parameters of PSCs with small molecular HTMs with dopants

HTM	Device structure	*µ*_h_ b) [cm^2^ V^−1^ s^−^]	HOMO [eV]	*V*_oc_ [V]	*J*_sc_ [mA cm^−2^]	FF [%]	PCE [%]	Ref.
Spiroa) doped with Li‐TFSI and TBP	FTO/cp‐TiO_2_/MAPbI_3‐_ *_x_*Cl*_x_*/HTM/Ag	–	–	1.07	21.5	67	15.4	13
Spiroa) doped with Li‐TFSI and TBP	ITO/PEIE/Y‐TiO_2_/MAPbI_3_/HTM/Au		5.22	1.13	22.75	75.01	19.3	43
Spiroa) doped with Li‐TFSI and TBP	ITO/ZnO/MAPbI_3_/HTM/Ag	–	–	1.03	20.4	74.9	15.7	26
Spiroa) doped with Li‐TFSI, TBP, and FK920	FTO/SnO_2_/(FAPbI_3_)_0.85_(MAPbBr_3_)_0.15_/HTM/Au	–	–	1.14	21.3	74	18.4	27
Spiroa) doped with Li‐TFSI and TBP	FTO/SnO_2_/MAPbI_3_/HTM/Au	–	–	1.11	23.27	67	17.21	44
Spiroa) doped with CuSCN	FTO/cp‐TiO_2_/MAPbI_3‐_ *_x_*Cl*_x_*/HTM/Ag	2.60 × 10^−3^	–	1.06	22.01	77	18.02	45
Spiroa) doped with CuI		1.80 × 10^−3^	–	1.06	21.52	73	16.67	
CzPAF‐SBF doped with Li‐TFSI	ITO/ZnO/MAPbI_3_/HTM/Au	2.18 × 10^−4^	−5.11	1.09	20.91	73.92	16.85	51
JY6 doped with Li‐TFSI and TBP	ITO/cp‐TiO_2_/MAPbI_3‐_ *_x_*Cl*_x_*/HTM/Ag	8.84 × 10^−4^	−5.21	1.066	21.39	81	18.54	52
F22 doped with Li‐TFSI and TBP	FTO/cp‐TiO_2_/MAPbI_3‐_ *_x_*Cl*_x_*/HTM/Ag	5.37 × 10^−4^	−5.27	1.05	21.26	79	17.71	53
F33 doped with Li‐TFSI and TBP		6.79 × 10^−4^	−5.32	1.11	21.01	79	18.48	
XPP doped with Li‐TFSI	ITO/SnO_2_/C_60_/MA_3_PbI_3_/HTM/Ag	1.60 × 10^−4^	−5.15	1.03	21.2	78.9	17.2	54
	ITO/SnO_2_/C_60_/FA_0.85_MA_0.15_Pb[I_0.85_Br_0.15_]_3_/HTM/Ag			1.11	23.14	74	19.1	
Cz‐OMeTAD doped with Li‐TFSI	FTO/SnO_2_/MAPbI_3_/HTM/Au	1.82 × 10^−3^	−5.27	1.14	22.26	71	17.81	56
CZ‐TA doped with Li‐TFSI and TBP	FTO/SnO_2_/C_60_‐SAM/MA_0.7_FA_0.3_PbI_3_/HTM/Au	1.65 × 10^−4^	−5.11	1.044	21.66	81	18.32	55
V950 doped with Li‐TFSI, TBP, and FK920	FTO/SnO_2_/C_60_/MAPbI*_x_*Cl_3−_ *_x_*/HTM/Au	1.90 × 10^−5^	−5.01	1.02	22.1	75	16.9	57
	FTO/SnO_2_/C_60_/FA_0.83_Cs_0.17_Pb(I_0.6_Br_0.4_)_3_/HTM/Au			1.15	18	66	13.7	

^a)^ Spiro: Spiro‐OMeTAD

^b)^ *µ*_h_: Hole mobility of HTMs.

Carbazole‐based HTMs have been proven promising in both organic light‐emitting diodes and organic solar cells (OSCs) and also a main kind of small molecule HTMs in planar PSCs.^58^, ^59^, ^60^, ^61^, ^62^, ^63^ This is owing to their excellent properties, such as cheap starting materials, strong electron‐donating property, suitable energy levels, special rigid structure, and large conjugated system, as well as the potential for structural modification by incorporation of different alkyl or functional groups into N atom or outer benzene ring of carbazole to tune optoelectronic properties, solubility, and molecular packing. Chen et al. reported a carbazole based small molecule material 1,3,6,8‐tetra(N,N‐p‐dimethoxyphenylamino)‐9‐ethyl‐carbazole(Cz‐OMeTAD, Figure 2), which was synthesized with a facial synthetic route utilizing inexpensive, air‐stable, and nontoxic raw materials.^59^ With suitable energy levels, high mobility, and conductivity with dopants, Cz‐OMeTAD based PSCs showed a high PCE of 17.81%, analogous to that of Spiro‐OMeTAD based devices. Yin et al. reported a carbazole‐based HTM named 4,4′,4″,4′″‐(9‐octylcarbazole‐1,3,6,8‐tetrayl) tetrakis(N,N‐bis(4‐methoxyphenyl)aniline) (CZ‐TA, Figure 2), which exhibited outstanding optoelectronic properties with facile synthesis process and low cost.^58^ The PSCs based on CZ‐TA HTM achieved a maximum PCE of 18.32% under reverse voltage scan and a steady‐state efficiency of 16.36%. Additionally, CZ‐TA did not require oxygen doping which was beneficial to improve device stability and its commercialization process. Although the synthesis routes of CZ‐TA and Cz‐OMeTAD were simple and low‐cost, they were still via cross‐coupling reactions that required transition metal catalysts, inert reaction conditions, and extensive product purification process in order to remove catalyst residue which in turn increased cost of the final product. Daskeviciene et al. reported a simple carbazole‐based conjugated enamine named V950 (Figure 2) with extremely simple route. The V950 HTM can be obtained by a single‐step enamine condensation reaction under ambient conditions from readily available commercial starting materials with water as the only by‐product, thus making product purification uncomplicated. Having a photovoltaic performance comparable to Spiro‐OMeTAD‐based devices, V950 HTM synthesized by enamine condensation chemistry offered a cost‐effective and up‐scalable HTMs synthesis route (one step synthesis, low cost, and simple purification).^60^

A series of donor–acceptor (D–A)‐type conjugated organic molecules have also been reported to replace amorphous Spiro‐OMeTAD HTMs, for their advantages, such as easy purification, tunable energy levels, high mobility and conductivity, and good film morphology. An oligothiophene derivative named DR3TBDTT (Figure 2) containing a backbone structure containing a benzodithiophene (BDT) electron‐donating central block and an ethylrhodanine electron‐withdrawing end group was synthesized by Gong and co‐workers.^64^ DR3TBDTT showed high hole mobility and matched HOMO energy level by modifying its alkyl groups, as a result, the highest PCE of 8.8% was achieved using DR3TBDTT adding a small amount of polydimethylsiloxane (PDMS) as the HTM without ion additive, comparable to 8.9% for Spiro‐OMeTAD doped with Li‐TFSI and TBP. Moreover, the absence of hydroscopic Li‐TFSI and the presence of hydrophobic oligothiophene could improve the device stability by resisting moisture invasion. To further understand the relationship between photovoltaic properties and molecular structure, Liu and co‐workers synthesized two D–A structure HTMs named DERDTS–TBDT and DORDTS–DFBT (Figure 2), consisting of dithienosilole (DTS) electron‐donating unit and 3‐alkyl rodanine electron‐withdrawing end group.^65^ With the electron donating property of alkylthienyl‐substituted benzo[1,2‐b:4,5‐b′]dithiophene (TBDT) and the electron withdrawing property of 5,6‐difluoro‐2,1,3‐benzothiadiazole (DFBT) as core, respectively, the two small molecules exhibited different energy levels and electronic properties. As a result, DERDTS–TBDT HTM‐based devices showed much higher photovoltaic efficiency than DERDTS–DFBT HTM‐based devices, which was ascribed to its matched energy levels and high hole mobility. This work showed that the energy‐level tuning of HTMs was a key factor to obtain high efficiency PSCs.

#### 2.1.2 p–i–n Inverted Structure

Poly(3,4 ethylenedioxythiophene):poly(styrenesulfonic acid) (PEDOT:PSS) was the most widely used HTM in inverted devices because of its high transparency, appropriate energy levels, and the compatibility with PSCs.^29^, ^66^, ^67^ However, it is prone to corrode indium tin oxide (ITO) and absorb the moisture from atmosphere because of its hygroscopic and acid nature, which degrade the devices performance and stability. Moreover, amine groups in perovskite precursors such as methylammonium iodide (MAI) can reduce the work function (WF) of PEDOT:PSS from ≈5.0 to 4.7 eV, thus lowering the maximum *V*_oc_ that can be achieved in PEDOT:PSS based PSCs.^68^ Recently, by modifying the chemical structure and optoelectronic properties through molecular engineering, several small molecule HTMs have been reported to replace PEDOT:PSS. Li and co‐workers et al. applied two novel small molecule HTMs with linear π‐conjugated structure, 4,4′‐bis(4‐(di‐p‐toyl)aminostyryl)biphenyl (TPASBP) and 1,4′‐bis(4‐(di‐p‐toyl)aminostyryl)benzene (TPASB) (Figure 2).^69^ Both of them possessed high hole conductivity and could promote the growth of perovskite film consisting of large grain size and less grain boundaries. With the effective hole extraction and transport in the TPASBP‐based and TPASB‐based devices, high efficiencies of 17.4% and 17.6%, respectively, were achieved. With similar linear π‐conjugated structure, two triarylamine based HTMs, named 4,4′‐(naphthalene‐2,6‐diyl)bis(N,N‐bis(4‐methoxyphenyl)aniline) (NTPA) and N4,N4,N4″′,N4″′‐tetrakis(4‐methoxyphenyl)‐[1,1′:4′,1″:4″,1″′‐quaterphenyl]‐4,4″′‐diamine (BTPA). (Figure 2), were synthetized and used in inverted PSCs.^70^ These HTMs showed increased efficiency compared to PEDOT:PSS and exhibited increased stability with lifetimes in excess of 500 h. Yang and co‐workers reported another π‐conjugated small molecule HTM with two triarylamine groups (4,4′‐cyclohexylidenebis[N,N‐bis(4‐methylphenyl) benzenamine] (TAPC), Figure 2) in inverted devices and obtained a high PCE up to 18.8%.^68^ Through thermal annealing treatment, a high hole conductivity and effective charge transfer were achieved contributing to the conjugated structure and partial crystallization of TAPC. The TAPC HTM also promoted perovskite film quality with large grain size and less unreacted PbI_2_ and stabilized the device due to its hydrophobic property. Also with the commonly used arylamine groups, Chen and co‐workers designed a HTM named Trux‐OMeTAD (Figure 2) consisting of a C3h truxene‐core with three arylamine groups and six hexyl side‐chains.^71^ This planar, rigid, and fully conjugated molecule rendered it high hole mobility and desired surface energy to the perovskite active layer. As a result, the Trux‐OMeTAD HTM based PSCs reached a high PCE of 18.6% with minimal hysteresis. Three novel triarylamine derivatives (TPACs: TPAC0M, TPAC2M, and TPAC3M, Figure 2) with the same conjugation scaffold but different numbers of methoxy units were designed as HTMs in inverted PSCs by Park et al.^72^ They all showed similar optical and electrical properties, but the TPAC3M HTM with three methoxy units exhibited better charge extraction and reduced recombination behaviors. The results showed that the Lewis base passivation effect of methoxy units could increase devices performance effectively. With methoxy groups and methylsulfanyl groups in the terminal substituents, respectively, Chen and co‐workers designed and synthesized two HTMs containing a tetraphenyl core and arylamine groups, named TPP‐OMeTAD and TPP‐SMeTAD (Figure 2).^73^ These two HTMs possessed marginal solubility in dimethylformamide (DMF) and could diffuse back into the crystal grain boundaries during the two‐step fabrication process of top perovskite layer, resulting in a perovskite/HTM heterojunction, as shown in Figure 3d. As a result, TPP‐SMeTAD, with deep HOMO level and strong Pb–S interaction with perovskite active layer, not only facilitated hole extraction but also passivated traps at both perovskite/HTM interface and the grain boundaries. Thus, the inverted devices based on TPP‐SMeTAD HTM reached a PCE of 16.6% with a negligible hysteresis. Huang et al. designed two groups of bulky steric diarylfluorene‐based HTMs with the triphenylamine (TPA) or 9‐phenylcarbazole (9‐NPC) as the arm and the octyl (C8) or the 9‐hexyl carbazole (C6Cz) as side line, namely, MC8‐TPA, MC8‐9‐NPC, MC6Cz‐TPA, and MC6Cz‐9‐NPC (Figure 2).^74^ All these smooth, amorphous, and hydrophobic HTMs films exhibited high hole conductivity and were beneficial to the growth of perovskite film. Consequently, these new HTM‐based devices yielded less carrier loss and higher PCE than that of PEDOT:PSS‐based devices. All photovoltaic parameters of perovskite solar cells based dopant free small molecular HTMs are listed in **Table** **2**.

**Table 2:** Photovoltaic parameters of PSCs with small molecular HTMs without dopants

HTM	Device structure	*µ*_h_ a) [cm^2^ V^−1^ s^−1^]	HOMO [eV]	*V*_oc_ [V]	*J*_sc_ [mA cm^−2^]	FF [%]	PCE [%]	Ref.
DR3TBDTT + PDMS	FTO/cp‐TiO_2_/MAPbI_3‐_ *_x_*Cl*_x_*/HTM/Au	≈10^−4^	−5.39	0.95	15.3	60	8.8	61
DERDTS–TBDT	ITO/cp‐TiO_2_/MAPbI_3‐_ *_x_*Cl*_x_* /HTL/MoO_3_/Ag	1.00 × 10^−4^	−5.09	1.05	21.2	72.8	16.2	62
TPASBP	ITO/HTM/MA_3_PbI_3_/PC_61_BM/Al	2.73 × 10^−4^	−5.00	1.04	20.7	80	17.4	66
TPASB		1.65 × 10^−3^	−5.49	1.05	20.8	80	17.6	
NAPT	ITO/HTM/MA_3_PbI_3_/PC_61_BM/Ag	1.10 × 10^−3^	−5.27	1.02	17.4	77.3	13	67
BTPA		4.70 × 10^−4^	−5.20	0.98	17.4	70.8	12.1	
TAPC	ITO/HTM/MA_3_PbI_3_/PC_61_BM/Ag	4.99 × 10^−4^	−5.5	1.04	22.25	80.22	18.6	65
Trux‐OMeTAD	ITP/HTM/MAPbI_3_/PC_61_BM/ZnO/Al	3.60 × 10^−3^	−5.28	1.02	23.2	79	18.6	68
TPAC3M	ITO/HTM/MA_3_PbI_3_/PC_61_BM/ZnO/Al	1.10 × 10^−5^	−4.96	1.00	22.79	78	17.54	69
TPP‐SMeTAD	ITO/HTM/MA_3_PbI_3_ + HTM/PC_61_BM/ZnO/Al	7.40 × 10^−5^	−5.18	1.07	20.15	77	16.6	70
MC8‐TPA	ITO/HTM/MA_3_PbI_3_/PC_61_BM/LiF/Al	–	−5.35	0.88	20.20	72	12.8	71
MC8‐9‐NPC		–	−5.51	0.90	20.80	74	13.85	
M6Cz‐TPA		–	−5.38	0.87	20.71	69	12.43	
M6Cz‐9‐NPC		–	−5.53	0.89	20.10	73	13.06	

^a)^ *µ*_h_: Hole mobility of HTMs.

### 2.2 Polymers

#### 2.2.1 n–i–p Inverted Architecture

Motivated by the goal of fabricating cost‐effective, high‐efficiency, and stable PSCs, polymeric HTMs with higher hole mobility than dopant‐free Spiro‐OMeTAD have been employed. Poly(3‐hexylthiophene‐2,5‐diyl) (P3HT, Figure 5), profiting from its low synthesis cost and dopant‐free feature, has been widely used in organic photovoltaic devices as well as PSCs.^75^, ^76^ At an early stage, Bi et al. compared the photovoltaic properties of HTM/(CH_3_NH_3_)PbI_3_/TiO_2_ solar cells using Spiro‐OMeTAD, P3HT, and 4‐(diethylamino)‐benzaldehyde diphenylhydrazone as HTMs.^77^ The devices with P3HT HTM showed a lower efficiency of 4.5% than Spiro‐OMeTAD‐based devices. The major reason was that P3HT has a rather flat molecular structure and the thiophene units may be in close contact with the perovskite surface, which resulted in a faster recombination of the separated charges. Afterward, Abbas et al. achieved a remarkable performance (PCE ≈ 13.7% and *V*_oc_ ≈ 0.96 V) using P3HT as HTM by optimizing its thickness and a sequential vapor deposited perovskite film as active layer.^78^ This work pointed that an optimized HTM thickness and a pin‐hole free active layer morphology were crucial to obtaining high PCEs.

The hole mobility of pristine P3HT is generally lower (≈10^−4^ cm^2^ V^−1^ s^−1^) than that of doped Spiro‐OMeTAD, therefore, efforts have been dedicated to enhance hole mobility via doping strategies. For example, Liu et al. reported a P3HT HTM, doped with an Li‐TFSI and TBP, enhancing the ordering of the polymer chains of P3HT and achieving two orders improvement of hole conductivity.^76^ Moreover, single‐walled carbon nanotubes (SWNTs) and graphdiyne (GD) were also used as dopants to enhance the hole conductivity of P3HT.^79^, ^80^ Heo et al. introduced a layered sandwich‐type architecture including a 3D nanocomposite and bilayer architecture, with perovskite as light harvester and different polymers such as poly‐[2,1,3‐benzothiadiazole‐4,7‐diyl[4,4‐bis(2‐ethylhexyl)‐4H‐cyclopenta[2,1‐b:3,4‐b′]dithiophene‐2,6‐diyl]], (poly‐[[9‐(1‐octylnonyl)‐9H‐carbazole‐2,7‐diyl]‐2,5‐thiophenediyl‐2,1,3‐benzothiadiazole‐4,7‐diyl‐2,5‐thiophenediyl]) (PCDTBT), and poly‐triarylamine (PTAA, Figure 5) as HTMs.^81^ Among these HTMs, devices based on PTAA HTM showed highest PCE up to 12%. When compared with Sprio‐OMeTAD‐based devices, PTAA‐based devices exhibited higher *V*_oc_ and FF because of the lower *R*_s_ and larger WF of later. However, when the mesoporous TiO_2_ layer was absent, the device performance was extremely poor (PCE ≈ 0.4%) for the generated charge carriers that were recombinated and could not be effectively collected from the too thick perovskite overlayer. Later on, the same group used a mixed solvent of γ‐butyrolactone and dimethylsulphoxide (DMSO) followed by toluene drop‐casting to form an extremely uniform and dense perovskite layer via a CH_3_NH_3_I–PbI_2_–DMSO intermediate phase, as a result, the planar devices with compact TiO_2_ ETM and PTAA HTM reached a PCE of 14.4% but with a large hysteresis.^15^ Then they reported a planar architecture device utilizing Zn_2_SnO_4_ (ZSO) nanoparticles as ETM and PTAA as HTM.^82^ Although this device still showed hysteresis in the current density–voltage (*J*–*V*) curves measured with reverse and forward scan, it was smaller than those of the TiO_2_‐based planar device mentioned above due to the higher charge collecting ability of ZSO ETM. Though hole conductivity of PTAA (≈1 × 10^−2^ – 1 × 10^−3^ cm^2^ V^−1^ s^−1^) is higher than P3HT and pristine Spiro‐OMeTAD, additives are often doped into it to further improve its conductivity.^83^ To thoroughly get rid of the negative effect of additives, alternative HTMs with excellent electrical properties have been reported in recent years. Donor–acceptor (D–A) type conducting polymers are candidate alternatives due to their unique and tunable optical and electrical properties, such as energy levels, low bandgap, high mobility, and good film formability.^84^ Kim and co‐workers reported a novel D–A polymeric HTM based on benzo [1,2‐b:4,5:b′]dithiophene (BDT) and 2,1,3‐benzothiadiazole (BT), named RCP, composed of P‐OR and P‐R as illustrated in Figure 5.^85^ P‐OR had high hole mobility (≈10^3^ cm^2^ V^−1^ s^−1^), however, its HOMO energy level was deeper than that of CH_3_NH_3_PbI_3_, which was unfavorable for hole transferring from perovskite to HTM. By combining P‐OR with P‐R, RCP which had reasonably deep HOMO energy level was obtained. With these superior electronic properties and hydrophobicity, dopant‐free devices showed a high PCE of 17.3% and maintained the initial efficiency for over 1400 h at 75% humidity. However batch‐to‐batch variations may exist in the synthesis of random copolymers. To avoid this issue, the same group later reported a well‐defined structure BDT–BT based D–A type conducting homopolymer HTM comprising tetraethylene glycol (TEG), named as PTEG (Figure 5).^86^ BDT and BT units rendered PTEG with a deep HOMO energy level and high hole mobility. The TEG side chains increased solubility and improved the contact between perovskite and HTM. Using SnO_2_ ETM with high electron mobility and PTEG HTM in a planar PSC, a high PCE of 19.8% was achieved which is one of the highest values among n–i–p type planar‐PSCs reported to date. In addition to charge transport function, the traps passivation effect of HTMs could reduce charge recombination at perovskite/HTM interface. Cai and co‐workers reported a series of carbazole and BT based D–A copolymers with different lengths of alkoxy side‐chains grafted on the BT unit, as HTMs in regular PSCs.^87^ Although the alkoxy sidechains may reduce the π–π stacking structural order and affect charge transport of HTMs effectively, the methoxy units could suppress charge recombination at perovskite/HTM interface. As a result, devices using the copolymer with methoxy side‐chains on the BT unit (named PCDTBT1, Figure 5) as HTM reached a high PCE up to 19.1%.

**Figure 4:**
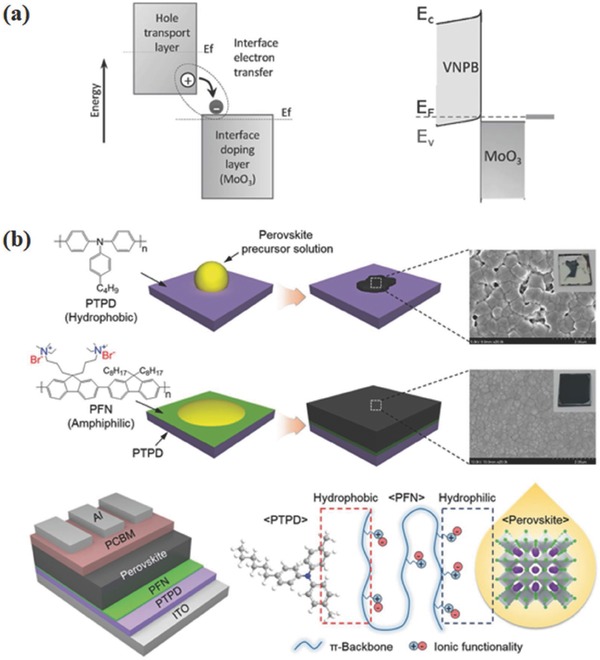
a) Schematic of interface doping: ground‐state electron transfer occurs from the hole transport layer to the interface material (MoO_3_), thereby enhancing the hole carrier density throughout the thin HTM. Reproduced with permission.^88^ Copyright 2016, John Wiley and Sons. b) Schematic illustrations of perovskite film formation on organic HTMs with and without the interfacial compatibilizer as well as amphiphilic interaction of PFN between PTPD and perovskite layers. Reproduced with permission.^104^ Copyright 2017, John Wiley and Sons.

**Figure 5:**
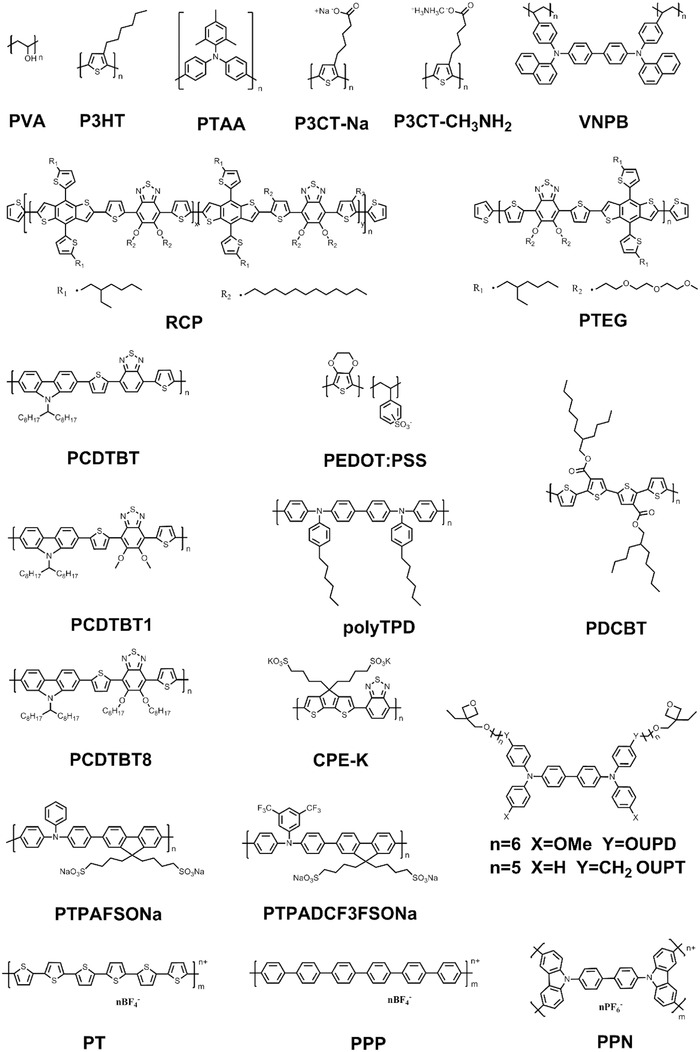
Molecular structures of polymeric HTMs.

Another strategy to enhance photovoltaic performance of PSCs is employing polymer HTMs/metal oxides bilayers to realize remote doping. Xu et al. employed an arylamine derivative (N4,N4‐Di(naphthalen‐1‐yl)‐N4,N4′‐bis(4‐vinylphenyl)biphenyl‐4,4′‐diamine) (VNPB, Figure 5) cross‐linked film coupled with MoO_3_ as HTM.^88^ The double‐layer HTM not only provided a thermally stable and solvent‐resistant protection to perovskite active layer, but also provided a stable and efficient doping process by ground‐state electron transfer between VNPB/MoO_3_ interfaces, which could increase hole density in HTM layer, as shown in **Figure** **4**a. With MoO_3_ interface doping layer, the photovoltaic performance, especially FF increased significantly. Recently, Hou and co‐workers adopted Ta doped WO*_x_* combined with polythiophene polymer, poly[5,5′‐bis(2‐butyloctyl)‐(2,2′‐bithiophene)‐4,4′‐dicarboxylate‐alt‐5,5′‐2,2′‐bithiophene] (PDCBT) (**Figure** **5**) as bilayer HTM.^89^, ^90^ The result showed that after Ta was incorporated into the WO*_x_* host lattice, its conductivity was enhanced by almost one order of magnitude. Similar to VNPB/MoO_3_ HTM, the PDCBT/Ta‐WO*_x_* bilayer also achieved a larger hole density via transferring electrons from the polymer to the metal oxides. The combination of high conductivity of Ta‐WO*_x_* and charge‐transfer doping fostered the formation of selective but low resistance contacts, which benefited hole transfer. As a result, a highest PCE of 21.2% among PSCs with ionic dopant‐free HTMs was achieved. All photovoltaic parameters of n‐i‐p structure PSC based polymeric HTMs are listed in **Table** **3**.

**Table 3:** Photovoltaic parameters of n–i–p structure PSCs with polymeric HTMs

HTM	Device structure	*µ*_h_ a) [cm^2^ V^−1^ s^−1^]	HOMO [eV]	*V*_oc_ [V]	*J*_sc_ [mA cm^−2^]	FF [%]	PCE [%]	Ref.
P3HT	FTO/cp‐TiO_2_/MAPbI_3_/HTM/Ag	–	–	0.96	21.76	65.3	13.7	75
P3HT doped with LiTFSI and TBP	ITO/TiO_x_/MAPbI_3‐_ *_x_*Cl*_x_*/HTM/Ag	6.40 × 10^−2^	–	0.98	19.1	66.3	12.4	73
P3HT‐SWNTs/PMMA	ITO/ cp‐TiO_2_/mp‐Al_2_O_3_/MAPbI_3‐_ *_x_*Cl*_x_*/HTM/Ag	–	−4.80	1.02	22.71	66	15.3	76
P3HT doped with GD	ITO/cp‐TiO_2_/mp‐TiO_2_/MAPbI_3_/HTM/Au	–	−4.90	0.941	21.7	71.3	14.58	77
PTAA	ITO/ cp‐TiO_2_/mp‐TiO_2_/MAPbI_3_/HTM/Au	4.00 × 10^−3^	−5.20	0.997	16.5	72.7	12	78
PTAA	FTO/ cp‐TiO_2_/mp‐TiO_2_/MAPb(I_1‐_ *_x_*Br*_x_*)_3_/HTM/Au	–	–	1.12	19.65	76	16.72	79
PTAA	PEN/ITO/ZSO/ MAPbI_3_/HTM/Au	–	–	1.05	21.6	67	15.3	80
PTAA doped with LiTFSI and TBP	FTO/cp‐TiO_2_/MAPbI_3_/HTM/Au	–	–	1.1	20.1	78	17.2	81
RCP	FTO/SnO_2_/MAPbI_3_/HTM/Au	3.09 × 10^−3^	−5.41	1.08	21.9	75	17.3	82
PTEG	FTO/SnO_2_/Cs‐perovskite/HTM/Au	1.64 × 10^−3^	−5.40	1.14	22.5	77	19.8	83
PCDTBT1	ITO/TiO_2_/PC_61_BM/MAPbI_3_/HTMs/MoO_3_/Au	–	−5.38	1.10	22.2	18.2	19.9	76
Cross‐linked VNPB/MoO_3_	FTO/cp‐TiO_2_/PC_61_BM/MAPbI_3_/HTM/Au	–	−5.40	1.11	19	81	16.5	85
PDCBT/Ta‐WO*_x_*	ITO/C_60_‐SAM /SnO_x_/PC_61_BM/FA_0.83_MA_0.17_Pb_1.1_Br_0.50_I_2.80_/HTM/Au	–	−5.30	1.17	22.7	80	21.2	86

^a)^ *µ*_h_: Hole mobility of HTMs.

#### 2.2.2 p–i–n Inverted Architecture

As mentioned above, the n–i–p structure planar PSCs usually suffer notorious hysteresis. To further understand the origin of hysteresis, Heo et al. studied the regular and inverted structure simultaneously (fluorine tin oxide (FTO)/TiO_2_/CH_3_NH_3_PbI_3_/PTAA:TBP+Li‐TFSI/Au and ITO/PEDOT:PSS/CH_3_NH_3_PbI_3_/PC_61_BM/Au).^83^ The inverted planar PSCs exhibited higher PCE, more free hysteresis, and better stability than the regular solar cells. Among these improved properties, the reduced *J*–*V* hysteresis could be attributed to a more balanced electron flux (*J*_e_) and hole flux (*J*_h_) owing to the better electron extraction in the [6,6]‐phenyl C61‐butyric acid methyl (PC_61_BM) ester electron conductor than in TiO_2_. PEDOT:PSS (Figure 5) is one of the most successful HTMs in the inverted (p–i–n) PSCs owning to its good film forming property, high transmissivity, and matched WF.^91^, ^92^ Snaith and co‐workers used and compared PEDOT:PSS, NiO, and V_2_O_5_ HTMs in inverted planar PSCs.^32^ Among these HTMs, a solid perovskite absorber layer exhibiting over 95% surface coverage was achieved on PEDOT:PSS HTM. Consequently, the inverted PSCs with low‐temperature HTM achieved a PCE up to 10% on glass substrates and over 6% on flexible polymer substrates. As PEDOT:PSS is sustainable to polar solvent wash, such as DMF or DMSO, most of inverted devices adopted PEDOT:PSS as HTM underneath the perovskite layer.^91^, ^93^, ^94^ However, the low WF of PEDOT:PSS caused a relatively small *V*_oc_ of around 0.90 V in these inverted planar heterojunction devices, compared to the typical *V*_oc_ of >1.05 V in the regular PSCs. In conclusion, improving the crystallinity, morphology, and surface coverage of the perovskite layer on the underlayer HTM and reducing mismatched energy levels between HTM and perovskite layer are all crucial factors to further increase the performance of inverted planar PSCs. Therefore, modification of PEDOT:PSS/perovskite interface is also important to fully unveil the potential of inverted PHJ PSCs. To further enhance the charge selectivity of PEDOT:PSS, Malinkiewicz et al. used a thin layer of poly(N,N′‐bis(4‐butylphenyl)‐N,N′‐bis(phenyl)benzidine) (polyTPD, Figure 5) as the electron blocking layer, as a result, the device performance increased significantly.^92^, ^95^ However, due to poor wetting of perovskite precursor processed from polar solvents (DMF or DMSO) on the hydrophobic polymer (polyTPD) surface, this type of bilayer structure was only compatible for vacuum‐deposited or sequential deposition PSCs. To deposit perovskite active layer by one‐step solution method, Cao *et al*. introduced two new conjugated copolymers with polar functional groups on side chains, named PTPAFSONa and PTPADCF_3_FSONa (Figure 5), as hole selective layers (HSLs) on PEDOT:PSS layer.^96^ HSLs with high surface energy and hydrophilic nature offered full coverage and high crystalline perovskite layer by a simple one‐step solution process. With the deep HOMO level and high lowest unoccupied molecular orbital (LUMO) level, insertion of PTPADCF_3_FSONa could reduce interfacial charge recombination and increase *V*_oc_ significantly. As a result, high‐performance CH_3_NH_3_PbI*_x_*Cl_3−_ *_x_* based PSCs with a PCE of 16.6% (*V*_oc_: 1.07 V) and CH_3_NH_3_Pb(I_0.3_Br_0.7_)*_x_*Cl_3−_ *_x_* based cells with a PCE of 10.3% (*V*_oc_: 1.34 V) were realized. Similarly, Chen and co‐workers used cross‐linked N,N′‐bis(4‐(6‐((3‐ethyloxetan‐3‐yl)‐methoxy)‐hexyloxy)phenyl)‐N,N′‐bis(4‐methoxyphenyl)biphenyl‐4,4′‐diamine (C‐QUPD) and N,N′‐bis(4‐(6‐((3‐ethyloxetan‐3‐yl)methoxy))‐hexylphenyl)‐N,N′‐diphenyl‐4,4′‐diamine (C‐OTPD) (Figure 5 **)** as HSLs between PEDOT:PSS and perovskite.^37^ C‐QUPD and C‐OTPD with high LUMO levels could block electrons transferring from perovskite to HTM and suppress the electron–hole recombination at perovskite/HTM interface, as a result, an increase of *V*_oc_ from 0.77 to 0.95 V and 0.99 V and PCE from 9.93% to 10.87% and 13.06%, respectively. Yu and co‐workers used a PEDOT:PSS and a graphene oxide composite layer (PEDOT:GO) as HTM and achieved a highly efficient and stable inverted PSCs.^97^ PEDOT:GO HTM exhibited higher electrical conductivity and a higher WF than PEDOT:PSS, and could enhance crystallinity of the perovskite crystal and suppressed leakage current simultaneously, which delivered an increased PCE from 14.95% to 18.09%.

However, PEDOT:PSS is prone to corrode ITO and absorb the moisture from atmosphere because of its hygroscopic and acid nature, which degrades the device's performance and stability. Moreover, amine groups in perovskite precursors such as MAI can reduce the WF of PEDOT:PSS from ≈5.0 to 4.7 eV, thus lowering the maximum *V*_oc_ that can be achieved in PEDOT:PSS based PSCs.^69^ Therefore, various new p‐type polymers are designed and synthetized. Heeger and co‐workers first reported an organic polyelectrolyte, poly[2,6‐(4,4‐bis‐potas‐siumbutanylsulfonate‐4H‐cyclopenta‐[2,1‐b;3,4‐b′]‐dithiophene)‐alt‐4,7‐(2,1,3‐benzothiadiazole)] (CPE‐K, Figure 5) as HTM in inverted PSCs.^38^ The excellent wettability of perovskite precursor solution on the CPE‐K layer led to a uniform perovskite film with complete surface coverage and superior hole selectivity for facilitating hole transport from perovskite to the ITO anode. As a result, the device with CPE‐K exhibited higher device efficiency, over 12%, than that of the device fabricated with widely used PEDOT:PSS. Furthermore, the CPE‐K‐based device showed improved air stability due to the neutral pH nature of CPE‐K. Similarly, Li et al. reported another water soluble polyelectrolyte P3CT‐Na (Figure 5) as HTM in inverted structure PSCs.^98^ With matchable energy level and good wettability with perovskite precursor, P3CT‐Na could transfer hole and block electron effectively to the anode even with a large thickness up to 52 nm. Later, the same group used P3CT‐CH_3_NH_2_ to replace P3CT‐Na as HTM to break up the strong aggregation of P3CT‐Na.^99^ The devices based on P3CT‐CH_3_NH_2_ HTM showed a greatly improved average PCE from 16.9 to 18.9%. Huang and co‐workers reported a series of conductive polymers, including poly(p‐phenylene) (PPP), polythiophene, and poly(4,40‐bis(N‐carbazolyl)‐1,10‐biphenyl) (Figure 5), and explored their application as HTMs in inverted PSCs. PPP, with WF of −5.31 V, could increase the *R*_rec_ and improve the device *V*_oc_ to 1.05 eV.^100^ Later, another work of the same group further proved that devices based on high WF HTM exhibited high *R*_rec_ and low charge recombination rate, thus resulting in high *V*_oc_.^101^ Chiang and co‐workers formed a solvent‐resistant polymerized HTM by thermal curing styrene‐functionalized 9,9‐diarylfluorene‐based triaryldiamine monomer N^2^,N^2^,N^7^,N^7^‐tetrakis(4‐methoxyphenyl)‐9,9‐bis(4‐((4‐vinylbenzyl)oxy)phenyl)‐9H‐fluorene‐2,7‐diamine (VB‐DAAF). With the good energy level alignment, the polymerized VB‐DAAF film could transport holes and block electrons effectively. Consequently, the best‐performing cell applying polymerized VB‐DAAF HTM reached PCE of 15.17%, which was higher than that of PEDOT:PSS based devices.^102^ Despite the hydrophilic HTM enable form of pin‐hole free and full coverage perovskite film, their hygroscopic nature has a detrimental effect on device stability. Therefore, Zhao et al. used only poly‐TPD as HTM and deposited perovskite film on it by a two‐step sequential method. The poly‐TPD HTM based devices exhibited high performance up to 15.3% benefiting from the large crystallites and full coverage of perovskite film on poly‐TPD compared to that on PEDOT:PSS HTM.^103^ Lee and co‐workers used an amphiphilic conjugated polyelectrolyte, poly[(9,9‐bis(3′‐(N,N‐dimethylamino)propyl)‐2,7‐fluorene)‐alt‐2,7‐(9,9‐dioctylfluorene)] (PFN, Figure 5) as an interfacial compatibilizer between HTM/perovskite interface.^104^ In this work, poly(N,N′‐bis(4‐butylphenyl)‐N,N′‐bis(phenyl)benzidine) (PTPD), whose HOMO was well matched with valence band level of CH_3_NH_3_PbI_3_, was used as HTM, but with low wettability of the perovskite precursor solution. The introduction of amphiphilic PFN on the PTPD surface considerably solved this problem and uniform perovskite films were fabricated (Figure 4b). Huang and co‐workers grew CH_3_NH_3_PbI_3_ film on a wide range of wetting and nonwetting HTMs, including polyvinyl alcohol (Figure 5), PEDOT:PSS, cross‐linked N4,N4′‐bis(4‐(6‐((3‐ethyloxetan‐3‐yl)methoxy) hexyl)phenyl)‐N4,N4′‐diphenylbiphenyl‐4,4′‐diamine (C‐OTPD), PTAA, and (N‐9′‐heptadecanyl‐2,7‐carbazole‐alt‐5,5‐(4′,7′‐di‐2‐thienyl‐2′,1′,3′‐benzothiadiazole)) (PCDTBT, Figure 5).^105^ With the increased nucleus spacing and the facilitated grain boundary migration in grain growth, the charge recombination in perovskite film on nonwetting HTMs was reduced. As a result, high stabilized device efficiencies of 18.3% and 17.8% were obtained, respectively, based on tetrafluoro‐tetracyanoquinodimethane (F4‐TCNQ) doped PTAA and C‐OTPD HTMs. Later, Zhou and co‐workers used graphene oxide (GO)/PTAA bilayer as HTM in inverted PSCs, to combine the UV‐stability of GO and the ability of PTAA to improve perovskite films quality. The bilayered HTM based devices showed a high PCE of 15.7% and 17.2%, respectively, on flexible and rigid substrates and an outstanding light‐soaking stability, retaining almost 90% of its original efficiency after continuous illumination for 500 h at 100 mW cm^−2^.^106^

For organic HTMs, Spiro‐OMeTAD and PEDOT:PSS are two commonly used materials, but with plenty of shortcomings such as expensive, extra oxygen doping process, acid, and hydrophilic feature. Though a number of new organic HTMs have emerged as mentioned above, there is still room for further improvements. However, we believe that for further developments of PSCs performance, deeper understanding of device physics such as charge recombination, extraction, and transport mechanisms should be moved forward. In addition, organic molecular designing and engineering should be explored to expand the function of HTMs such extra light absorption, reducing hysteresis, and promote device stability. Moreover, reduction in the cost and synthesis complexity is necessary for commercial applications in the industry. All photovoltaic parameters of p‐i‐n structure PSC based polymeric HTMs are listed in **Table** **4**.

**Table 4:** Photovoltaic parameters of p–i–n structure PSCs with polymeric HTMs

HTM	Device structure	*µ*_h_ a) [cm^2^ V^−1^ s^−1^]	HOMO [eV]	*V*_oc_ [V]	*J*_sc_ [mA cm^−2^]	FF [%]	PCE [%]	Ref.
PEDOT:PSS	ITO/HTM/MAPbI_3_/PC_61_BM/Au	–	–	1.1	20.9	79	18.2	81
PEDOT:PSS	ITO/HTM/MAPbI_3_/ICBA/C_60_/BCP/Al	–	–	0.97	15.7	80.1	12.2	88
PEDOT:PSS	glass/FTO/HTM/MAPbI_3‐_ *_x_*Cl*_x_*/PC_61_BM/TiO*_x_*/Al		−5.20	0.94	15.8	66	9.8	90
	PET/ITO/HTM/MAPbI_3‐_ *_x_*Cl*_x_*/PC_61_BM/TiO*_x_*/Al			0.88	14.4	51	6.4	
PEDOT:PSS	ITO/HTM/MAPbI_3_/PC_61_BM/C_60_/BCP/Al	–	–	0.99	19.6	79.3	15.4	91
PEDOT:PSS	FTO/HTM/MAPbI_3‐_ *_x_*Cl*_x_*/PC_61_BM/Al	9.30 × 10^−3^	−5.18	0.94	22.4	83	17.47	92
PEDOT:PSS/poly TPD	ITO/HTM/MAPbI_3_/PC_61_BM/Au	–	−5.30	1.05	16.12	67	12.04	89
PEDOT:PSS/poly TPD	ITO/HTM/MAPbI_3_/PC_61_BM/Au	–	−5.40	1.09	18.2	75	14.8	93
PEDOT:PSS/PTPADCF_3_FSONa	ITO/HTM//MAPbI_3‐_ *_x_*Cl*_x_*/PC_61_BM/PN_4_N/Ag	3.20 × 10^−5^	−5.39	1.07	20.4	75.9	16.6	94
PEDOT:PSS/c‐QUPD	ITO/HTM/MAPbI_3‐_ *_x_*Cl*_x_*/PC_61_BM/Al	4.18 × 10^−6^	−5.12	0.99	18.07	73	13.06	37
PEDOT:GO	ITO/HTM/perovskite/PCBM/ZnO/Ag	‐	−5.42	1.02	21.55	82.3	18.09	95
CPE‐K	ITO/HTM/MAPbI_3‐_ *_x_*Cl*_x_*/PC_61_BM/Al	‐	−4.90	0.89	20.1	70	12.51	38
P3CT‐Na	ITO/HTM/MAPbI_3‐_ *_x_*Cl*_x_*/PC_61_BM/C_60_/Al	7.70 × 10^−4^	−5.26	1.07	21.4	73.2	16.6	96
P3CT‐CH_3_NH_2_	ITO/HTM/MAPbI_3_/PC_61_BM/C_60_/BCP/Al	1.08 × 10^−5^	–	1.09	22.2	81.0	19.6	97
PPP	ITO/HTM/MAPbI_3_/PC_61_BM/C_60_/BCP/Ag	–	−5.31	1.02	21.7	75.4	16.7	98
PCT	ITO/HTM/MAPbI_3_/PC_61_BM/C_60_/BCP/Ag	–	−5.40	1.01	21.4	76.4	16.5	99
PTPD/PFN	ITO/HTM/MAPbI_3_/PC_61_BM/Al	–	−5.40	1.103	20.98	73.6	17.04	100
PTAA doped with F4‐TCNQ	ITO/HTM/MAPbI_3_/PC_61_BM/C_60_/BCP/Al	–	–	1.07	22.0	76.8	18.1	101
c‐OTPD				1.05	22.4	75.6	17.8	
rGO/PTAA	Glass/ITO/HTM MAPbI_3_/PC_61_BM/BCP/Ag		−5.22	1.09	20.3	77.7	17.2	102
	PEN/ITO/HTM MAPbI_3_/PC_61_BM/BCP/Ag			1.09	19.2	75	15.7	

^a)^ *µ*_h_: Hole mobility of HTMs.

## 3 Electron Transport Materials

### 3.1 Small Molecules

#### 3.1.1 p–i–n Inverted Structure

**Figure 6:**
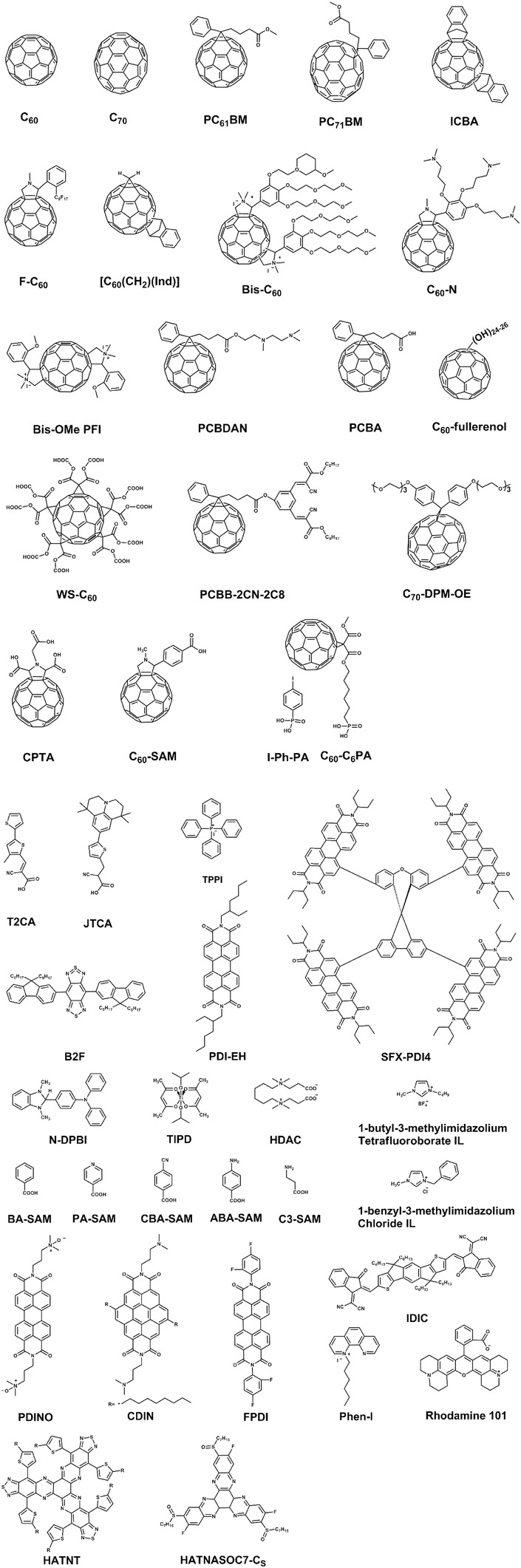
a) Molecular structures of fullerene‐based small molecular ETMs or cathode interfacial modifiers. b) Molecular structure of non‐fullerene‐based small molecular ETMs or cathode interfacial modifiers.

*Electron Transport Layer*: Fullerene and its derivatives have been widely employed in PSCs as ETMs since first reported by Chen and co‐workers.^29^ They fabricated planar heterojunction CH_3_NH_3_PbI_3_ perovskite/C_60_ (**Figure** **6**a) or C_60_ derivatives (such as PC_61_BM ester (Figure 6a) and indene‐C60 bisadduct (ICBA, Figure 6a)) solar cells in which CH_3_NH_3_PbI_3_ acted as both light absorber and hole conductor. Applying PC_61_BM and ICBA with higher LUMO level instead of C_60_ elevates the magnitude of *V*_oc_ from 0.55 to 0.65 or 0.75 V. Despite their PCE was limited by the magnitude of short‐circuit (*J*_sc_), which could be improve by adjusting the thickness, morphology, and topography of perovskite film. Despite the optimized PCE in this work was still low (3.9%), they pave the way of fullerene materials used in inverted planar PSCs and directed that further optimization of perovskite film can enhance their photovoltaic performance. Subsequently, plenty works based on C_60_, PC_61_BM, ICBA, or [6,6]‐phenyl C_71_‐butyric acid methyl ester (PC_71_BM, Figure 6a) have been reported to improve the PCE of fullerene‐based PSCs.^31^, ^107^ Intrinsic properties of fullerene materials and the interaction at perovskite/fullerene interface, such as electron mobility, energy level alignment, and the passivation effect on perovskite trap states, are key factors influencing device performance. Liang et al. systematically studied three fullerene materials, ICBA, PC_61_BM, and C_60_ as ETMs in inverted devices.^108^ The PCEs of the fullerene‐based PSCs clearly followed the trend of increased electron mobility in the fullerene layer. This result demonstrated that high‐mobility fullerenes could effectively promote charge dissociation/transport and photovoltaic performance in a PSC. Moreover, fullerene materials can passivate interfacial traps and reduce interfacial energy barrier between perovskite and fullerenes, which can elucidate the minor hysteresis in these fullerene‐based devices. Some open issues such as the hysteresis in *J*–*V* curve when scanned at different directions, the light‐soaking requirement to enhance PCE under continuous illumination, and the long‐term operational instability limited the development of perovskite technology. To address these problems, reduced graphene oxide (rGO) was introduced into PC_61_BM ETM of inverted planar PSCs.^109^ On one hand, rGO not only increased the conductivity of PC_61_BM which was responsible to the enhanced *J*_sc_ and FF, but also reduced the surface traps and passivated the perovskite surface which resulted in higher *V*_oc_ and reduced light soaking effect. On the other hand, rGO can stabilize perovskite/PC_61_BM interface and improve the device operation stability in ambient conditions. Such effect also existed in the inverted PSCs with 2,6‐dimethoxypyridine(2,6‐Py) doped PC_61_BM as ETM, in which the 2,6‐Py doped PC_61_BM showed improved conductivity and mobility for efficient electron extraction and transport.^110^ The 2,6‐Py could also passivate the Lewis acid trap states of undercoordinated Pb ions existing on the perovskite film surface. The dual‐function treatment significantly enhanced the device performance from 15.53% to 19.41%. In addition to PCE, the reproducibility and air stability were simultaneously improved and benefited from the reduced trap states at PC_61_BM/perovskite interface. Kuang et al. doped PC_61_BM ETM with GD in inverted PSCs.^111^ After doping, PC_61_BM:GD ETM showed better coverage on perovskite layer and increased electron mobility, which resulted in 28.7% enhancement of PCE compared to the PC_61_BM based devices. The high electron mobility and effective traps passivation effect of PC_61_BM make it a good ETM in inverted PSCs, further enhance is possible to form better energetic coherence between perovskite and ETM. Xue and co‐workers designed a 56‐electron dihydromethano/indene fullerene [C_60_(CH_2_)(Ind)] (Figure 6a) with a shallow LUMO level to replace PC_61_BM.^112^ Benefiting from the well matched energy level with the conduction band of the perovskite layer, a high *V*_oc_ of 1.13 V was obtained which outperformed that of commonly used PC_61_BM ETM based devices. This study showed that fullerenes with higher LUMO could reduce the nonradiative recombination losses and maximize the voltage output. Xing et al. substituted commonly used PC_61_BM ETM with a series of fullerene derivatives, various phenyl groups of the diphenylmethanofullerene (DPM) moiety decorated with oligoether (OE) chains.^113^ The electron‐rich OE chains could both passivate perovskite trap states and reduce the WF of the metal cathode, as a result, the interfacial properties such as energy levels, charge carrier mobilities, surface energy, and dipole layer feature could be tuned by controlling the number of OE chains. By this strategy, devices used the monoadduct fullerene derivative C_70_‐DPM‐OE (Figure 6a), as ETM achieved a PCE of 16% much higher than that of PC_61_BM‐based devices.

A deep study on the photodegradation mechanism of inverted planar PSCs with PC_61_BM ETM was carried on by Akbulatov and co‐workers.^114^ They found PC_61_BM could absorb some degradation products of perovskite, which corroded the top metal electrode and caused rapid degradation of perovskite. Meanwhile, they also applied a soluble perylene diimide (PDI) derivative, PDI‐EH (Figure 6b), as ETM in inverted planar PSCs. The PDI‐EH compact film can isolate photoactive layer from metal electrode physically, preventing CH_3_NH_3_I (the decomposition product of perovskite) reacting with it but back with PbI_2._ As a result, the operation stability of devices with PDI‐EH ETM was improved significantly. Cheng et al. also synthesized a PDI tetramer‐based 3D molecular material, termed SFX‐PDI4 (Figure 6b) as nonfullerene ETM.^115^ Profiting from the 3D conformations, the SFX‐PDI4 film exhibited isotropic charge transport ability and weak molecular aggregation. When implied in inverted planar PSCs, a rival PCE compared to PC_61_BM‐based device was obtained. Considering the simple synthesis and purification processes, the finely tuned PDI‐based ETMs paved a way to further improve the efficiency and stability of planar PSCs. In addition to the PDI‐based nonfullerene ETMs, Zhao and co‐workers designed a serious of Hexaazatrinaphthylene (HATNA) based ETMs with high charge conductivity.^116^ To improve its solution processability and to tune the energy levels, the HATNA‐based ETMs were peripherally functionalized with alkylsulfanyl chains of different lengths and with different sulfur oxidation states. PSCs with the derivate HATNASOC7‐C_s_ (Figure 6b) as ETM exhibited a high PCE up to 17.6% and improved stability due to the hydrophobic ETM and its trap‐passivation function. Similarly, Wang et al. proposed a novel n‐type sulfur‐containing HATNA‐based small molecule, hexaazatrinaphtho[2,3‐c][1,2,5]thiadiazole (HATNT, Figure 6b), with high electron mobility and matched energy levels as an ETM in planar heterojunction PSCs.^117^ The HATNT ETM could suppress the charge recombination at the perovskite/ETM interface more effectively than PC_61_BM ETM, thus achieving a high PCE of 18.1% which was much higher than the device using PC_61_BM as ETM. Zhu and co‐workers synthesized a benzobis(thiadiazole)‐based small molecule called B2F (Figure 6b) and applied it as an electron extraction material in inverted perovskite devices.^118^ With matched LUMO levels with the CB of perovskite layer and good adhesion on the perovskite surface, B2F could effectively extract electron from perovskite active layer. Combined with C_60_/BCP bilayer, the device with B2F ETM could reach a PCE of 17.18%. All photovoltaic parameters of p‐i‐n structure PSC based small molecular ETMs are listed in **Table** **5**.

**Table 5:** Photovoltaic parameters of p–i–n structure PSCs with small molecular ETMs

ETM	Device structure	*µ*_e_ b) [cm^2^ V^−1^ s^−1^]	LUMO [eV]	*V*_oc_ [V]	*J*_sc_ [mA cm^−2^]	FF [%]	PCE [%]	Ref.
C_60_	ITO/PEDOT:PSS/MAPbI_3_/ETM/BCP/Al	–	−4.50	0.55	9.02	61	3.0	29
PC_61_BM			−3.90	0.60	10.32	63	3.9	
ICBA			−3.73	0.58	10.03	58	3.4	
PC_71_BM	ITO/PEDOT:PSS/MAPbI_3_/ETM/Ca/Al	–	–	1.05	19.98	78	16.31	103
PC_61_BM	ITO/PEDOT:PSS/MAPbI_3_/ETM/Al	1.0	−4.20	0.91	10.8	76	7.4	104
ICBA	ITO/PEDOT:PSS/MAPbI_3_/ETM/Bis‐C_60_/Ag	6.90 × 10^−3^	−3.60	0.95	11.27	75	8.06	105
PC_61_BM		6.10 × 10^−2^	−3.80	0.89	18.85	80	13.37	
C_60_		1.6	−3.90	0.92	21.17	80	15.44	
PC_61_BM doped with rGO	ITO/PEDOT:PSS/MAPbI_3‐_ *_x_*Cl*_x_*/ETM/PFNa)/Ag	0.495c)	–	0.85	22.92	65.8	12.82	106
	ITO/PEDOT:PSS/MAPbI_3_/ETM/PFN/Ag			0.942	23.52	65.5	14.51	
PC_61_BM doped with 2,6‐Py	ITO/NiO_x_/MAPbI_3_/(2,6‐Py)/ETM/PEI/Ag	3.80 × 10^−3^	–	1.09	23.14	77	19.41	107
PC_61_BM:GD	ITO/PEDOT:PSS/MAPbI_3‐_ *_x_*Cl*_x_*/ETM/C_60_/Al	5.32 × 10^−4^	–	0.969	23.4	65.4	14.8	108
C_60_(CH_2_)(Ind)	ITO/NiO/DEA/MAPbI_3_/C_60_(CH_2_)(Ind)/PN4N/Ag	3.00 × 10^−3^	−3.66	1.13	20.4	80	18.1	109
C_70_‐DPM‐OE	ITO/PEDOT:PSS/MAPbI_3‐_ *_x_*Cl*_x_*/ETM/Ag	3.30 × 10^−4^	−3.86	0.97	21.9	75	16	110
PDI‐EH	ITO/PEDOT:PSS/MAPbI_3_/ETM/Ag	–	–	0.846	19.7	61	10.3	111
SFX‐PDI4	FTO/NiMgLiO/(MAPbI_3_)_0.85_(MAPbCl_3_)_0.15_/ETM/Ti(Nb)O*_x_*/Ag	1.80 × 10^−4^	−3.94	1.08	19.9	71.4	15.3	112
HATNASOC7‐C_s_	ITO/NiO*_x_*/MAPbI_3_/ETM/Ag	5.13 × 10^−3^	−3.92	1.08	20.73	78.6	17.62	113
HATNT	ITO/PEDOT:PSS/MAPbI_3_/ETM/LiF/Al	1.73 × 10^−2^	−4.00	1.07	21.83	77.8	18.1	114
B2F	ITO/P3CT‐Na/MAPbI_3_/ETM/C_60_/BCP/Ag	4.13 × 10^−3^	−4.02	1.052	20.63	79.15	17.18	115

^a)^ PEN: Polyethylene naphthalate

^b)^ Electron mobility of ETMs

^c)^ Electron conductivity of ETMs [σ_e_ (mS cm^−1^)].

*Cathode Buffer Layer*: In the inverted planar heterojunction PSCs, the electronic structure and energy alignment at the metal electrode/ETM interface are essential factors that affect the device performance. Various interfacial engineering approaches have focused on inserting a cathode buffer layer between metal electrode and ETM. To form ohmic contact and reduce interface barrier between the common used PC_61_BM ETM and metal cathode, several cathode buffer materials including LiF, Ca, BCP, TiO*_x_*, and ZnO have been reported.^12^, ^29^, ^32^, ^49^, ^119^, ^120^ However, many drawbacks of them such as the need of vacuum deposition process, instability in ambient condition, the difficulty to control thickness due to its insulated property, and the complicated operational process restricted their employment. To overcome these problems, some organic semiconductors such as fullerene derivatives and other small molecules are employed as cathode buffer layers. Liang et al. applied a bis‐adduct fullerene surfactant (Bis‐C_60_, Figure 6a) to align the energy levels at PC_61_BM/cathode interface and to utilize stable metals such as Ag as the top electrodes.^121^ Later, the same group formed a robust and efficient cathode interface by combining a novel fullerene derivative with a perfluoroalkyl side‐chain (F‐C_60_, Figure 6a) with their previously reported Bis‐C_60_ surfactant.^122^ Besides the enhanced performance, the ambient stability was simultaneously improved due to the hydrophobic F‐C_60_ and the reduced moisture penetration into devices. Because the amine functional groups can create interface dipoles and shift WF of metal electrode, several amine functionalized fullerenes can act as efficient cathode buffer layers. A fulleropyrrolidine‐based interfacial modification layer with amine substituent (C_60_‐N, Figure 6a) was inserted between the silver electrode and PC_61_BM ETM.^123^ This C_60_‐N interlayer could enhance *R*_rec_, increase electron extraction rate, and prolong free carrier lifetime, as a result, a significant improvement of PCE from 7.5% to 15.5% was achieved. Another fullerene amine interlayer ([6,6]‐phenyl‐C_61_‐butyric acid 2‐((2 (dimethylamino)‐ethyl) (methyl)amino)ethyl ester (PCBDAN), Figure 6a) was used by Xie et al. to reduce the interface barrier between ETM and metal electrode and also to resist the moisture penetration.^124^ As a result, the utilization of PCBDAN interlayer could effectively increase both the device performance and stability.

In addition to the fullerene‐based materials, Min et al. employed a solution‐processed perylene‐diimide (PDINO, Figure 6b) as cathode interfacial layer between PC_61_BM ETM and the top Ag electrode.^125^ PDINO could form a highly qualitative contact with the top Ag electrode and give homogeneous and smooth films with a good collection probability, which facilitated the efficient electron extraction and prevented the diffusion of Ag ions to the semiconductor interface. As a result, an improved PCE of 11.3%, which was higher than that of PC_61_BM/ZnO/Ag device with high stability, was obtained. As a successful electron transfer material in polymer solar cells, an alcohol soluble titanium chelate TIPD (titanium (diisopropoxide) bis(2,4‐pentanedionate), Figure 6b) was also used as an dipole interlayer to form an ohmic contact with the negative electrode.^126^ With the enhanced charge extraction rate and the suppressed charge recombination after the insertion of TIPD, the devices based on one‐step and two‐step synthesized CH_3_NH_3_PbI_3_ active layer all showed enhanced photocurrent as well as the power conversion efficiency. Sun and co‐workers introduced a halide ions‐based interlayer, tetraphenylphosphonium iodide (TPPI, Figure 6b), as an interfacial n‐dopant to modify the cathode interface between PC_61_BM and Al, which resulted in reduced contact resistance at the cathode interface.^127^ Consequently, the *J*_sc_ and FF were improved thus achieving a high device performance. Zwitterions with the positive and negative charges on the same molecule could lower the WF of electrodes by the formation of surface dipoles and reduce charge transfer resistance. Therefore, zwitterions could also be used as an efficient electron‐selective interfacial layer to align the energy levels between the fullerene ETM and the top metal. For instance, Sun et al. applied rhodamine 101 (Figure 6b) as the electron‐collection interfacial layer between PC_61_BM and the Ag electrode of PSCs.^128^ As a result, the PC_61_BM/rhodamine 101/Ag based device exhibited an increased performance mainly due to the improvement of FF. To explore the role of these organic molecules in the charge transfer at the metal/ETM interface and the chemical degradation processes of PSCs, Ciro and co‐workers also inserted rhodamine 101 as an interlayer between Ag and fullerene derivatives (PC_61_BM and PC_70_BM) ETMs.^129^ The rhodamine 101 not only could reduce nonradiative recombination at ETM/cathode interface and enhance charge transfer toward the metal cathode, but also could inhibit the ingress of moisture toward the perovskite and simultaneously prevent the migration of iodide that potentially could react with the silver cathode. Consequently, the dual‐functional interlayer both enhanced the PCE and operational stability in p–i–n planar PSCs. Zhu et al. employed a carboxylate functionalized zwitterionic carboxylate 3,3′‐(hexane‐1,6‐diylbis(dimethylammonionediyl))dipropanoate (HDAC) (Figure 6b) as interlayer between PC_61_BM/Ag.^130^ The device performance especially FF increased from 72% to 80% contributing to the formation of interface dipole and elimination of interfacial potential barrier. Recently, Hu and co‐workers synthesized a simple Phenanthroline derivative Phen‐I (Figure 6b) as cathode interlayer between metal electrode and PC_61_BM ETM.^131^ Phen‐I displayed a dual function property, including lowering the WF of the metallic cathode to increase electron extraction and doping into ETM to enhance the conductivity. As a result, a considerable enhancement in PCEs, from 10.70% to 18.13% in inverted PSCs, was obtained. All photovoltaic parameters of p‐i‐n structure PSCs with cathode interfacial modifiers are listed in **Table** **6**.

**Table 6:** Photovoltaic parameters of p–i–n structure PSCs with cathode interfacial modifiers

Modifier	Device structure	LUMOa) [eV]	*V*_oc_ [V]	*J*_sc_ [mA cm^−2^]	FF [%]	PCE [%]	Ref.
LiF	ITO/PEDOT:PSS/MAPbI_3_/PC_61_BM/modc)/Al	−4.30b)	0.866	20.7	78.3	14.1	116
Ca	ITO/PEDOT:PSS/MAPbI_3_/PC_71_BM/mod/Al	–	1.05	19.98	78	16.31	117
BCP	ITO/PEDOT:PSS/MAPbI_3_/PC_61_BM/mod/Al	−3.50	0.60	10.32	63	3.9	29
Ti(Nb)O*_x_*	FTO/NiMgLiO/MAPbI_3_/PC_61_BM/Ti(Nb)O*_x_*/Ag	−4.00	1.072	20.21	74.8	16.2	12
TiO*_x_*	FTO/PEDOT:PSS/MAPbI_3_ *_x_*Cl*_x_*/PC_61_BM/mod/Al	−4.00	0.94	15.8	66	9.8	90
ZnO	ITO/PEDOT:PSS/MAPbI_3_ *_x_*Cl*_x_* /PC_61_BM/mod/Al	–	0.97	20.5	80.1	15.9	45
Bis‐C_60_	FTO/PEDOT:PSS/MAPbI_3‐_ *_x_*Cl*_x_* /PC_61_BM/mod/Ag	–	0.92	17.5	73	11.8	118
F‐C_60_ and Bis‐C_60_	ITO/PEDOT:PSS/MAPbI_3_ *_x_*Cl*_x_*/PC_61_BM/mod/Ag	–	0.97	21.2	75.4	15.5	119
C_60_‐N	ITO/PEDOT:PSS/FA_1‐_ *_x_*MA*_x_*PbI_3_/PC_61_BM/mod/Ag	−3.90	–	20.5	–	15.5	120
PCBDAN	FTO/NiO/MAPbI_3_/PC_61_BM/mod/Ag	−4.10b)	1.08	20.71	77	17.2	121
PDINO	ITO/PEDOT:PSS/ MAPbI_3_ *_x_*Cl*_x_*/PC_61_BM/mod/Ag	−3.63	0.95	18.8	78.5	14.0	122
TIPD	FTO/PEDOT:PSS/MAPbI_3_/PC_61_BM/mod/Al	−3.90	0.89	22.57	64.5	12.95	123
TPPI	ITO/PEDOT:PSS/MAPbI_3_ *_x_*Cl*_x_*/PC_61_BM/mod/Al	–	0.90	19.7	73.0	13.0	124
Rhodamine 101	ITO/PEDOT:PSS/MAPbI_3‐_ *_x_*Cl*_x_*/PC_61_BM/mod/LiF/Ag	–	1.01	17.9	73.0	13.2	125
Rhodamine 101	ITO/NiO*_x_*/MAPbI_3_/PC_70_BM/mod/Ag	−4.30b)	1.04	21.6	74.0	16.6	126
HDAC	ITO/P3CT‐Na/MAPbI_3_/PC_61_BM/mod/Ag	−3.82b)	1.075	19.78	80.48	17.10	127
Phen‐I	ITO/NiO*_x_*/FA_0.3_MA_0.7_PbI_2.7_Cl_0.3_/PC_61_BM/mod/Ag	−3.62	1.07	23.14	77.98	19.27	128

^a)^ The LUMO energy level of the interfacial modification layer

^b)^ Work function of modified metal cathode

^c)^ Mod: Modifier at ETM/metal cathode.

#### 3.1.2 n–i–p Regular Architecture

*Surface Modifier*: In regular PSCs, TiO_2_ is a widely used ETM and most high efficiency devices are based on mosoporous TiO_2_ ETM. However, in the planar structure, compact TiO_2_ ETM suffers several drawbacks such as low electrical conductivity, UV unstability, and anomalous hysteresis in current–voltage curves at different voltage scans. To address these issues, fullerene derivatives or nonfullerene small molecules are self‐assembled on compact TiO_2_ surface. They could improve the electron dipole between ETM and perovskite layer and facilitate electron transfer at this interface. Abrusci et al. first reported a C_60_‐SAM (Figure 6a) layer on mesoporous TiO_2_ as an effective electron acceptor.^132^ Afterward, the same team also used C_60_‐SAM to modify the surface of compact TiO_2_ layer.^133^ With accelerated electron transfer and reduced nonradiative recombination at ETM/perovskite interface, the fullerene modified devices achieved a PCE up to 15.7% with significantly reduced hysteresis. Also as a common ETM, PC_61_BM was employed to modify the low‐temperature solution‐processed TiO*_x_* in regular PSCs.^134^ Due to the efficient charge extraction ability of PC_61_BM modifier, improved PCE and negligible hysteresis were both obtained. To prevent PC_61_BM solubilizing in polar solvent such as DMF or DMSO, two‐step procedure was implemented with a thermally evaporated PbI_2_. A water‐soluble fullerene derivative (WS‐C_60_, Figure 6a) was applied to re‐engineer the surface of the PC_61_BM layer and to improve the wettability of PbI_2_ solution by Brabec and co‐workers. They simplified the perovskite film forming process and enabled a two‐step full‐solution method.^93^ In addition to PC_61_BM, some fullerene derivatives with different functional groups have been used as interfacial modified materials between compact TiO_2_ and perovskite layer. For instance, a hydroxylated C_60_, fullerenol (Figure 6b), was introduced to modify TiO_2_ in n–i–p structure for its water solubility and excellent electron conductivity.^135^ The insertion of fullerenol dramatically facilitated the charge transport and decreased the interfacial resistance which resulted in greatly improved device performance. Dong et al. inserted a [6,6]‐phenyl‐C61‐butyric acid (PCBA, Figure 6b) monolayer between compact TiO_2_ and perovskite.^136^ The PCBA monolayer acted as a hole blocking layer which reduced trap site density on TiO_2_ and inhibited the charge recombination at the TiO_2_/perovskite interface, subsequently a high *V*_oc_ of 1.16 V and 80% improvement of PCE were obtained. Moreover, PCBA could anchor on TiO_2_ by forming chemical bond between TiO_2_ and carboxyl group, thus prevent being washed when depositing perovskite via one step solution process. A triblock fullerene derivative (PCBB‐2CN‐2C8, Figure 6b) as a cathode modification layer on TiO_2_ was synthesized by Li and co‐workers.^137^ PCBB‐2CN‐2C8 passivated the deep trap states on the TiO_2_ surface which reduced the charge recombination loss at the ETM/perovskite interface and avoided the rapid degradation caused by the UV sensitive TiO_2_ surface. Besides fullerene and its derivatives, ionic liquid (IL) with high large electrical conductivity, high charge mobility, and superior optical transparency can also modify the surface of TiO_2_. Yang and co‐workers used 1‐butyl‐3‐methylimidazolium tetrafluoroborate ionic liquid (Figure 6b) on TiO_2_ layer to form a surface dipole, and a high PCE up to 19.62% was obtained.^138^ Moreover, the IL modified ETM could suppress ion migration in the perovskite layer and charge accumulation at the interfaces, balance the hole flux at the anode, and benefit the growth of high quality perovskite absorber, as a consequence, the notorious hysteresis was completely eliminated.

In addition to TiO_2_, multiple other low‐temperature solution processed metal oxide ETMs such as ZnO, SnO_2_, and WO*_x_* were reported for regular planar PSCs. To further promote their device efficiency, a thin layer of fullerenes or other dipole small molecules were inserted at ETMs and perovskite interface. To enhance the crystalline property of perovskite film, Zuo and co‐workers modified ZnO ETM with 3‐aminopropanioc acid (C3‐SAM).^139^ With the permanent dipole moment of C3‐SAM, a better energy level alignment between ETM and perovskite layer was achieved. Similarly, Azmi et al. combined two high‐dipole‐effect molecules (T2CA and JTCA, Figure 6b) self‐assembled monolayer respectively with ZnO ETM.^140^ Performing as surface wetting controlling layers and electric dipole layers, the SAMs improved the quality of PbI_2_ and final perovskite layers, as well as the charge extraction ability at ZnO/perovskite interface. To enhance the thermal stability of CH_3_NH_3_PbI_3_ on the ZnO ETM surface, Zhang and co‐workers used a thin layer of PC_61_BM to passivate ZnO surface and to prevent its direct contact with perovskite.^141^ With good band edge alignment and high electron mobility, SnO_2_ is also an excellent ETM in regular PSCs. To improve electron transport and passivate traps at ETM/perovskite interface, PC_61_BM was also used as interfacial modifier on SnO_2_ ETM.^142^ Another major drawback of regular planar PSCs is the presence of hysteresis. To solve this issue, Hou et al. incorporated a mixed fullerene functionalized self‐assembled monolayers (SAMs) including I‐Ph‐PA and C_60_‐C6‐PA (Figure 6b) on the WO*_x_* ETM, and fabricated a hysteresis‐free planar regular device with a high PCE near to 15%.^143^ Although a variety of SAMs have been developed to enhance device performances of PSCs, the effects of chemical interactions between perovskite films and the substrates were neglected. To distinguish the role of chemical interactions from other contributing factors, such as energy level alignment and surface induced morphological variations, Zuo et al. used benzoic acid, 4‐pyridinecarboxylic acid (PA), 3‐aminopropanoic acid (C3), 4‐aminobenzoic acid, and 4‐cyanobenzoic acid (Figure 6b) anchored onto a SnO_2_ ETM to form different chemical interactions.^144^ The results proved that chemical interactions were the more predominant factor than energy level alignment theory governing the interfacial optoelectronic properties. All photovoltaic parameters of n‐i‐p structure PSCs with cathode interfacial modifiers are listed in **Table** **7**.

**Table 7:** Photovoltaic parameters of n–i–p structure PSCs with cathode interfacial modifiers

Modifier	Device structure	LUMOc) [eV]	*V*_oc_ [V]	*J*_sc_ [mA cm^−2^]	FF [%]	PCE [%]	Ref.
C_60_‐SAM	FTO/cp‐TiO_2_/mp‐TiO_2_/moda)/MAPbI_3_ *_x_*Cl*_x_*/P3HT/Ag	−3.95	0.81	14.9	55.5	6.7	129
C_60_‐SAM	FTO/cp‐TiO_2_/mod/MAPbI_3‐_ *_x_*Cl*_x_*/Spirob)/Au	–	1.04	22.1	75	17.3	130
PC_61_BM	FTO/TiO*_x_*/mod/MAPbI_3_/Spirob)/Au	–	1.11	21.0	76.6	17.9	131
PC_61_BM/WS‐C_60_	ITO/TiO*_x_*/mod/MAPbI_3_/P3HT/MoO*_x_*/Al	−4.10	0.95	27.4	56.3	14.6	91
Fullerenol	ITO/TiO_2_/mod/MAPbI_3‐_ *_x_*Cl*_x_*/P3HT/MoO_3_/Ag	−4.27	0.95	20.91	71.5	14.69	132
PCBA	ITO/TiO_2_/mod/MAPbI_3_/Spirob)/Ag	−4.20	1.14	19.62	63	14.08	133
PCBB‐2CN‐2C8	ITO/TiO_2_/mod/MAPbI_3_/Spirob)/Au	−4.01	1.06	20.68	79.1	17.35	134
1‐butyl‐3‐methylimidazolium tetrafluoroborate ionic liquid (IL)	ITO/TiO_2_/mod/MAPbI_3_/PTAA/Au	−4.01d)	1.12	22.75	77	19.62	135
C_3_‐SAM	ITO/ZnO/mod/MAPbI_3_/Spirob)/MoO_3_/Ag	−3.52d)	1.07	22.51	65	15.67	136
T2CA	ITO/ZnO/mod/MAPbI_3_/Spirob)/Au	−4.24d)	1.13	21.72	76	18.82	137
JTCA	ITO/ZnO/mod/MAPbI_3_/Spirob)/Au	−4.03d)	1.09	21.34	73	17.07	
PC_61_BM	ITO/ZnO/mod/MAPbI_3_/Spirob)/Au	−3.90	1.08	22.80	77.3	19.0	138
CPTA + PbI_2_	ITO/ZnO/mod/MAPbI_3_/Spirob)/Au	–	1.114	22.36	81.13	22.2	180
PC_61_BM	FTO/SnO_2_/mod/MAPbI_3_/Spirob)/Au	–	1.12	22.16	75.8	19.12	139
C_60_‐C_6_‐PA + I‐Ph‐PA – SAMs	ITO/WO*_x_*/mod/perovskite/Spirob)/Ag	–	1.02	21.9	66.5	14.9	140
4‐pyridinecarboxylic acid (PA) – SAM	ITO/SnO_2_/mod/MAPbI_3_/Spirob)/Au	−4.17d)	1.10	22.03	77.4	18.77	141

^a)^ Mod: Modifier at metal oxides/perovskite interface

^b)^ Spiro: Spiro‐OMeTAD

^c)^ The LUMO energy level of the interfacial modification layer

^d)^ Work function of modified ETM.

**Figure 7:**
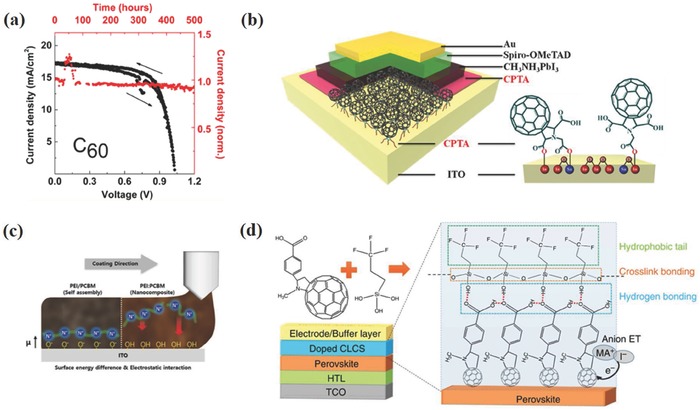
a) *J*–*V* characteristic of planar heterojunction PSCs with C_60_ ETM. Reproduced with permission.^28^ Copyright 2015, American Chemical Society. b) Schematic device structure of planar n–i–p PSCs with CPTA as the ETM and its covalent interaction with ITO. Reproduced with permission.^152^ Copyright 2017, John Wiley and Sons. c) Proposed scheme for the self‐assembly of the PEI in the PEI:PC_61_BM during the printing process. Reproduced with permission.^150^ Copyright 2017, John Wiley and Sons. d) Device structure of the perovskite planar heterojunction solar cells and schematic illustration for the cross‐linking of C_60_‐SAM with silane‐coupling agent. Reproduced under the terms of the Creative Commons CC BY license.^164^ Copyright 2016, Springer Nature.

*Electron Transfer Layer*: The commonly used TiO_2_ ETM usually needs high‐temperature sintering process to obtain high crystallization_._ Although the low‐temperature processed TiO_2_, ZnO, and CdSe nanoparticles have been developed as ETMs, the relevant devices showed a large hysteresis in the *J*–*V* curve and a pronounced decline of the average efficiency.^25^, ^26^, ^145^ Moreover, the complicated fabrication processes and difficulty in precise size control also hinder their application. To address these problems, fullerene‐based materials as alternatives to the inorganic ETMs are reported widely. Snaith and co‐workers employed a fullerene C_60_ compact layer instead of the commonly employed compact TiO_2_ in a regular n–i–p planar architecture device.^28^ Electron extraction was more efficient at C_60_/perovskite interface for the enhanced electronic coupling action, which led to a negligible hysteresis and highly stabilized PCE (**Figure** **7**a). However, as C_60_ was soluble in DMF and the inferior wettability of PbI_2_ solution on it, the perovskite layer must adapted two‐step method with evaporated PbI_2_ layer. Later on, the same group doped C_60_ film with 3‐dimethyl‐2‐phenyl‐2,3‐dihydro‐1H‐benzoimidazole (N‐DMBI) to tune its surface wettability. As a result, a void‐free perovskite film was formed via one‐step solution process on the improved wetting ETM surface.^146^ N‐DMBI dopant provided a higher conductivity and reduced the WF of C_60_ which enhanced electron extraction. When implying the doped C_60_ in FA_0.83_Cs_0.17_Pb(I_0.6_Br_0.4_)_3_ based solar cells, 80% of the “post burn‐in” efficiency after 650 h stressing under continuous full spectrum solar illumination in air without encapsulation was achieved. In addition to C_60_, Collavini et al. also used solution processed C_70_ (Figure 6a) as ETM in regular devices and developed a comparative and systematic study of C_60_‐based and C_70_‐based solar cells.^147^ Although C_60_ and C_70_ had nearly identical LUMO levels, C_70_ bore inferior electron mobility which was approximately two orders of magnitude lower than that of C_60_. And it could absorb sunlight over a broad region of the visible spectrum. The two intrinsic properties of C_70_ were detrimental when used as ETM in regular PSCs. Innovatively, by use of fullerene‐saturation approach which replaced DMF with a DMF fullerene‐saturated solution as the solvent of perovskite precursor, C_70_ film thickness and its solubility in perovskite processing solutions were well controlled. The C_60_‐based and C_70_‐based devices showed comparable PCE and the latter showed lower *J*_sc_ due to its higher sunlight absorption. Besides pristine fullerenes, Ryu et al. first introduced fullerene derivatives PC_61_BM ETM together with polyethyleneimine (PEI) interfacial modifier into n–i–p architecture.^148^ By a modified solvent engineering process employing a diethylether drip as an orthogonal solvent, a thick perovskite with 100% perfect surface coverage on PC_61_BM was fabricated. Optimization of the thickness of the PC_61_BM layer yielded an overall PCE of 15.3% but with severe hysteresis in the *J*–*V* curve when measured in the forward and reverse bias scans. To reduce the hysteresis in PEIE/PC_61_BM based devices, Kim and co‐workers used a bilayered FPI‐PEIE/PC_61_BM ETM (FPI‐PEIE: fulleropyrrolidinium iodide derivative (bis‐OMe FPI) with a very small amount of polyethyleneimine (PEIE)), yielding a promising PCE of 15.7% comparable to the value of the PEIE/PC_61_BM based PSCs but with negligible hysteresis.^149^ Modifying FPI‐PEIE surface with PC_61_BM was to simultaneously improve the perovskite crystal quality and charge extraction at FPI‐PEIE/CH_3_NH_3_PbI_3_ interface. The FPI‐PEIE interfacial layer could modify the energy level of ITO and facilitate electron transferring from PC_61_BM to ITO cathode. Moreover, the room‐temperature processability and decent electronic property of FPI‐PEIE/PC_61_BM ETM could also be employed in a flexible PSC yielding an impressive PCE of 10%. Except for the bilayered ETM, Lee et al. introduced single layered self‐assembled organic nanocomposites (SAONs) containing n‐type polyelectrolytes and fullerene derivatives (PEI:PC_61_BM) as a new ETM in regular PSCs.^150^ Driven by the surface energy difference of the SAONs components, a spontaneous vertical phase separation appeared and formed dual‐functional ETM containing an electron acceptor and surface WF modifier, as shown in Figure 7c. The dual‐functional ETM facilitated electron transferring at ETM/perovskite interface, as a result, a high PCE of 18.1% without hysteresis was achieved. With the vertical self‐assemble of organic nanocomposites, the ETM could be formed via one‐step solution process without precise controlling of the thickness of polyelectrolytes layer. Based on the same principle, Xie et al. introduced PCBDAN (Figure 6a) interlayer between PC_61_BM and ITO by one‐step solution process.^151^ PCBDAN self‐assembled on the ITO driven by the Lewis‐base interaction and close surface energy between PCBDAN and ITO. The insertion of self‐organized PCBDAN interlayer reduced the WF of ITO electrode and eliminated the interface barrier between ETM and electrode. These PSCs employing PC_61_BM and PCBDAN ETM reached an efficiency of 18.1% with almost free of hysteresis. The vertical phase separation of the complex including modifier layer and transport layer simplified the fabrication process and was compatible with the roll‐to‐roll processing on flexible substrates. An untreated fullerene derivate called C_60_ pyrrolidine tris‐acid (CPTA, Figure 6a) without any interfacial modified material was used as ETM by Wang and co‐workers.^152^ The uniform CPTA film was anchored onto the surface of ITO by the covalent interaction between carboxy and indium or tin atom (Figure 7b). As a result, the CPTA layer could avoid being washed by perovskite precursor and made solution‐processed active layer possible. Moreover, CPTA exhibited high electron mobility, matched energy levels and effective electron transfer between active layer and ETM. Consequently, CPTA‐based devices showed a high PCE of 18.39% on glass substrates with free hysteresis and remarkable long‐term stability, and a PCE up to 17% on flexible substrates with enhanced flexural strength.

In addition to the fullerene‐based ETMs, PDI with high electron mobility can also been employed in regular PSCs as a monolayer ETM. Huang and co‐workers selected fluorinated perylene diimide (FPDI) as with two fluorine groups (FPDI, Figure 6b) as ETM in regular planar devices.^153^ After the FPDI film was annealed in chloroform solvent vapor for 30 min, a dense and uniform perovskite film with high substrate coverage could be obtained. FF and PCE of the devices were significantly enhanced. The same group developed a N,N′‐bis(3‐(dimethylamino)propyl)‐5,11‐dioctylcoronene‐2,3,8,9‐tetracarboxdiimide (CDIN, Figure 6b) ETM for high performance regular devices.^154^ The discotic coronene diimide core with an expanded π‐conjugated plane along the short axis of PDI was favorable for enhanced π–π stacking in solid state, thus affording respectable charge carrier mobility. And the further functionalized with polar amine‐containing sidechains modulated its polarity for enhancing the crystal growth of atop perovskite layer. Zhang and co‐workers pioneered the use of a fused‐ring electron acceptor ((2,2′‐[(4,4,9,9‐tetrahexyl‐4,9‐dihydro‐s‐indaceno[1,2‐b:5,6‐b′]dithiophene‐2,7‐diyl) bis[methylidyne(3‐oxo‐1H‐indene‐2,1(3H)‐diylidene)]]bis‐propanedinitrile) (IDIC), Figure 6b) as ETM to replace TiO_2_ in regular PSCs.^155^ Due to the high mobility, suitable energy levels, the hydrophobicity, and incompatible wetting surface, IDIC‐based devices exhibited enhanced perovskite crystallinity and film quality in parallel to efficient electron extraction and transport at ETM/perovskite interface, as a result, a champion PCE of 19.1% was achieved. Moreover, solid‐state ionic‐liquids (ss‐IL) which have been mentioned as a modifier layer on TiO_2_ ETM are also promising candidates as ETMs in perovskite devices. Liu and co‐workers used a ss‐IL (1‐benzyl‐3‐methylimidazolium chloride, Figure 6b) as low‐temperature processed ETM.^156^ Benefiting from the outperformed properties of this ss‐IL, such as antireflection, high electron mobility, and suitable WF, the PCE of champion device based on CH_3_NH_3_PbI_3_ was raised up to 15.04% and that of flexible PSCs based on (HC(NH_2_)_2_PbI_3_)_0.85_(CH_3_NH_3_PbBr_3_)_0.15_ reached 16.09%. In addition, the ss‐IL ETM could reduce the electron trap‐state density of perovskite active layer and then effectively suppress hysteresis. All photovoltaic parameters of n‐i‐p structure PSC based small molecular ETMs are listed in **Table** **8**.

**Table 8:** Photovoltaic parameters of n–i–p structure PSCs with small molecular ETMs

ETM	Device structure	*µ*_e_ c) [cm^2^ V^−1^ s^−1^]	LUMO [eV]	*V*_oc_ [V]	*J*_sc_ [mA cm^−2^]	FF [%]	PCE [%]	Ref.
LT‐TiO_2_ a)	FTO/ETM/mp‐Al_2_O_3_/MAPbI_3‐_ *_x_*Cl*_x_*/Spirob)/Ag	6.80 × 10^−4^ d)	–	1.02	21.5	71	15.9	25
ZnO	ITO/ETM/MAPbI_3_/Spirob)/Ag	–	–	1.03	20.4	74.9	15.7	26
CdSe	ITO/ETM/MAPbI_3_/Spirob)/Ag	1.20 × 10^−5^	−4.40	0.99	17.4	67.9	11.7	142
C_60_	ITO/ETM/MAPbI_3_/Spirob)/Au	–	–	1.03	19.8	65	13.5	28
C_60_ doped with N‐DMBI	ITO/ETM/MAPbI_3‐_ *_x_*Cl*_x_*/Spirob)/Au	9.00 × 10^−3^ d)	–	1.06	23	75	18.3	143
C_70_	FTO/ETM/MAPbI_3_/Spirob)/Au	–	–	0.995	15.1	69	10.4	144
PEI/PC_61_BM	FTO/PEI/PC_61_BM/MAPbI_3_/PTAA/Au	–	−4.20	0.98	21.8	72	15.3	145
FPI‐PEIE/PC_61_BM	ITO/ETM/MAPbI_3_/Spirob)/Au	1.10 × 10^−4^	−3.95	1.10	19.5	73	15.7	146
PEI:PC_61_BM	FTO/ETM/MAPbI_3_/PTAA/MoO_3_/Au	–	−4.00	1.10	20.73	79	18.1	147
PC_61_BM:PCBDAN	ITO/ETM/MAPbI_3_/Spirob)/Au	–	−4.10	1.08	21.7	77.3	18.1	148
CPTA	ITO/ETM/MAPbI_3_/Spirob)/Au	5.40 × 10^−3^	−3.90	1.10	22.06	75.61	18.39	149
FPDI	TTO/ETM/MAPbI_3_/Spirob)/MoO_3_/Ag	1.10 × 10^−2^	−4.22	0.96	15.29	54.03	7.93	150
PEIE/CDIN	ITO/ETM/MAPbI_3_/Spirob)/Ag	5.24 × 10^−3^	−3.80	1.06	21.5	75	17.1	151
IDIC	ITO/ETM/MAPbI_3_(Cl)/Spirob)/Au	1.10 × 10^−3^	−3.90	1.08	23.0	77	19.1	152
1‐benzyl‐3‐methylimidazolium chloride ionic liquid (IL)	ITO/ETM/MAPbI_3_/Spirob)/Au	1.00 × 10^−3^	−4.32	1.00	20.55	73.2	15.04	153

^a)^ LT‐TiO_2_: Low temperature processed TiO_2_

^b)^ Spiro: Spiro‐OMeTAD

^c)^ Electron mobility of ETMs

^d)^ Electron conductivity of ETMs [σ_e_ (mS cm^−1^)].

### 3.2 Polymers

Huge amount of excellent organic polymers as ETMs or acceptors in OSCs has been implied in PSCs. Given the easy tunable optical, electronic, and electrical properties, the absence of acceptor polymer materials, flexible fabrication processes, better film formation property, and potentiality to donate extra current density, it is worth exploring and making use of ideal polymeric materials as ETMS in PSCs to improve the PSCs technology.

#### 3.2.1 n–i–p Regular Architecture

**Figure 8:**
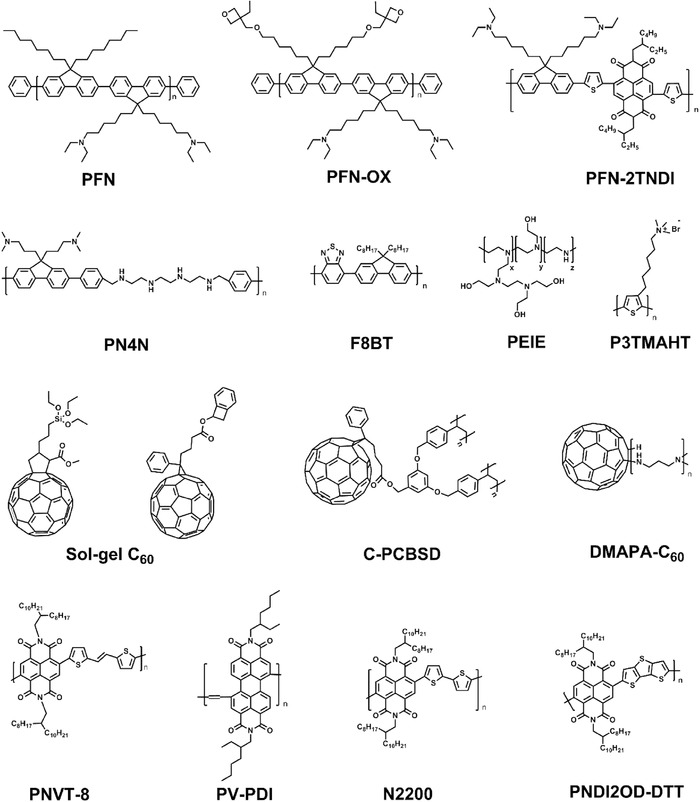
Molecular structures of polymeric ETMs.

In the regular structure, the surface modification strategy of TiO*_x_*, ZnO ETM, or ITO substrate has been demonstrated to achieve n‐type contact between the active layer and the electrode.^157^, ^158^ An inverted OSC with PFN (**Figure** **8**) as cathode interfacial layer had showed a high efficiency up to 9.2%, which demonstrated PFN was competent to transfer electron effectively.^31^ However, due to their solubility in polar solvent, the PFN‐based interfaces were not compatible in regular PSCs. To solve this discrepancy, Zhu and co‐workers used a compound with cross‐linkable conjugated polymer (PFN‐OX, Figure 8), which was solvent resistant and metal nanoparticles to form a hybrid ETM.^159^ With the interface dipole effect of amino groups in PFN‐OX:ZnO, an appropriate energy level alignment between the perovskite material and ITO electrode was achieved. Moreover, the compact, robust PFN‐OX:ZnO layer formed an optimized ohmic contact with minimum resistance and high charge selectivity. Compared with the high PCE, the cross‐linking concept introduced in this study is more valuable. A cross‐linkable [6,6]‐phenyl‐C61‐butyric styryl dendron ester (C‐PCBSD, Figure 8) was introduced into CH_3_NH_3_PbI*_x_*Cl_3‐_ *_x_* to enhance the crystallization and charge extraction ability of perovskite layer by Li et al.^160^ Afterward, the same group used the polymer but composited with large π‐conjugated GD to modify TiO_2_.^161^ After GD modification, the C‐PCBSD film exhibited a face‐on orientation which was favorable for growth and crystallization of the subsequent perovskite film and an improved electron mobility. The optimized interface and active layer quality are essential to efficiency improvement. In addition, the solvent resisted interface also made the interfacial erosion rate slower and improved device stability. Similarly, Tao et al. also used the same polymer (C‐PCBSD) to modify TiO*_x_*.^162^ Due to the matched energy level and high quality of the perovskite bulk film, a high *V*_oc_ over 1.1 eV was obtained. Wojciechowski et al. deposited cross‐linked fullerene derivatives containing a triethoxysilane moiety (N‐[3‐(triethoxysilyl)propyl]‐2‐carbomethoxy‐3,4‐fulleropyrrolidine), called sol–gel C_60_ (Figure 8) directly on FTO as a monolayer ETM.^163^ The polymerization technique will enable the fullerene ETMs solvent‐resistant and make the upscale of solution‐processed PSCs more possible.

#### 3.2.2 p–i–n Inverted Architecture

Although fullerene materials (typically PC_61_BM) are widely used ETMs in highly efficient inverted architecture PSCs, the thin fullerene layer cannot effectively protect perovskite from moisture damage in a humid environment. To address this issue, Bai et al. bonded cross‐linkable silane molecules trichloro(3,3,3‐trifluoropropyl)silan with hydrophobic functional groups onto C_60_‐substituted benzoic acid self‐assembled monolayer (C_60_‐SAM) as ETM in inverted perovskite devices, shown in Figure 7d.^164^ Such ETM prevented water molecules permeation and slowed the perovskite degradation down. The cross‐linking process and MAI as an effective n‐dopant for C_60_‐SAM both could improve its conductivity. The perovskite devices with the cross‐linked and doped C_60_‐SAM yielded a high efficiency of 19.5% without photocurrent‐hysteresis. In addition, the stability was significantly improved that 90% of the original PCE could be retained after exposing to an ambient environment for 30 d. The common used n‐type acceptors, e.g., naphthalenediimide (NDI)‐based or PDI‐based conjugated polymers in OSCs, can also be effective ETMs in planar PSCs. The first report to introduce organic polymer as ETM in inverted PSCs was from Wang and co‐workers who introduced nonfullerene‐based organic polymers, such as N2200, PNVT‐8, and PNDI2OD‐TT (Figure 8) as ETMs to replace PC_61_BM and obtained a rival PCE with PC_61_BM based devices.^165^ This work provides new direction to utilize organic polymers as a new class of ETM in inverted PSCs to further promote this technique. Zhao et al. reported a poly (9,9‐dioctyfluorene‐co‐benzothiazole) (F8BT, Figure 8) combined with C_60_ as ETM in inverted devices.^166^ A considerable enhancement of PCE from 7.9% in the absence of P8BT to 13.9% with F8BT/C_60_ ETM was achieved, and also with eliminated hysteresis. An aminofunctionalized copolymer with a conjugated backbone composed of fluorene, naphthalene diimide, and thiophene spacers (PFN‐2TNDI, Figure 8) ETM was reported by Sun and co‐workers.^167^ With the amines on the polymer side chains, not only the surface traps of perovskite were passivated but also the metal cathode's WF was reduced via forming desired interfacial dipole. Benefiting from the dual functionalities, surface recombination and interfacial resistance were reduced and therefore led high device efficiency. Moreover, owning to the good electron transport property of PFN‐2TNDI ETM, a wide range thickness of it was compatible to produce high efficiency device, facilitating the employment of large‐area coating method. Later, Guo and co‐workers showed a series of PDI based polymers (PX‐PDIs, Figure 8), which contain different copolymerized units (X), including vinylene (V), thiophene (T), selenophene (Se), dibenzosilole, and cyclopentadithiophene introduced as electron transporting layer (ETL) for p–i–n structured PSCs.^168^ With the deeper LUMO energy level, high electron mobility, and a better planar structure, PV‐PDI demonstrated best electron transfer property and the device with PV‐PDI as ETM showed highest PCE among these PDI based polymers.

Another strategy to optimize interfacial electronic property is to insert a cathode buffer layer. For instance, Xue and co‐workers introduced a new aminofunctionalized polymer (PN4N, Figure 8) as a cathode buffer layer in fullerene/perovskite (CH_3_NH_3_PbI*_x_*Cl_3‐_ *_x_*) planar heterojunction solar cells.^169^ The PN4N buffer layer reduced the contact resistance, suppressed the interfacial charge recombination, and also improved the electron transport property of the PSCs, thus resulting a remarkably enhanced PCE from 12.4% to 15.0%. In addition to modifying the WF of FTO or ITO cathode in regular devices, polyelectrolyte interlayers can also act as excellent modifiers to tune the WF of metal cathode in inverted structure devices.^148^, ^149^ Based on this principle, Zhang et al. inserted an ultrathin polyelectrolyte layer either ethoxylated PEIE (Figure 8) or poly [3‐(6‐trimethylammoniumhexyl)thiophene] (P3TMAHT, Figure 8) on top of PC_61_BM and beneath the Ag electrode.^170^ By the formation of surface dipoles, the WF of Ag electrode was reduced and thus reducing the energy barrier at the interface between the PC_61_BM and Ag. Both PEIE and P3TMAHE can improve the device performance and prove that the polyelectrolyte can be an excellent alternative choice as metal electrode modifier. Later on, the same team utilized an amine functionalized fullerene derivative (DMAPA‐C_60_, Figure 8) as a dipole layer to reduce the WF of the Ag electrode.^171^ The insertion of DMAPA‐C_60_ could reduce the injection barrier and nonradiative charge carrier recombination at the PC_61_BM/Ag interface, which significantly improved device performances. All photovoltaic parameters of n‐i‐p structure PSC based polymeric ETMs are listed in **Table** **9**.

**Table 9:** Photovoltaic parameters of PSCs with polymeric ETMs or modifiers of cathode

ETM	Device structure	*µ*_e_ a) [cm^2^ V^−1^ s^−1^]	LUMO [eV]	*V*_oc_ [V]	*J*_sc_ [mA cm^−2^]	FF [%]	PCE [%]	Ref.
PFN‐OX:ZnO	ITO/ETM/MAPbI_3_/Spiro/Au	–	−4.20	1.02	20.2	75	15.5	156
TiO_2_/C‐PCBSD:GD	FTO/ETM/MAPbI_3_/Spiro/Au	7.90 × 10^−4^	–	1.11	23.3	78	20.19	158
TiO_2_/C‐PCBSD	FTO/ETM/MAPbI_3_/Spiro/Au	≈10^−5^	−4.41	1.1	21.0	80	18.7	159
Sol–gel C_60_	FTO/ETM/MAPbI_3‐_ *_x_*Cl*_x_*/Spiro/Au	3.80 × 10^−4^	–	1.07	23.0	73	17.9	160
PCBCB		5.90 × 10^−3^	–	1.11	22.4	73	17.9	
Silane‐functionalized C_60_‐SAM	ITO/PTAA/MAPbI_3_/ETM/BCP/Cu	1.10 × 10^−2^	–	1.07	22.6	80.6	19.5	161
N2200	ITO/PEDOT:PSS/MAPbI_3‐_ *_x_*Cl*_x_*/ETM/ZnO/Cu	–	−3.93	0.84	14.7	66	8.15	162
PNVT‐8		–	−3.91	0.85	13.53	62	7.17	
PNDI2OD‐TT		–	−3.87	0.81	13.71	55	6.11	
F8BT	ITO/PEDOT:PSS/MAPbI_3‐_ *_x_*Cl*_x_*/ETM/C_60_/Al	≈10^−3^	−3.75	0.94	22.4	63.8	13.9	163
PFN‐2TNDI	ITO/PEDOT:PSS/MAPbI_3‐_ *_x_*Cl*_x_*/ETM/Ag	4.80 × 10^−4^	−3.84	0.98	21.9	78	16.7	164
PV‐PDI	FTO/PEDOT:PSS/MAPbI_3_/ETM/Al	3.00 × 10^−3^	−4.05	0.931	16.6	65.6	10.41	165
Modifier of metal cathode			*Φ* b) [eV]					
PN4N	ITO/PEDOT:PSS/MAPbI_3‐_ *_x_*Cl*_x_*/PC_61_BM/mod/Al	–	–	1.00	20.61	72.5	15	166
PEIE	ITO/PEDOT:PSS/MAPbI_3‐_ *_x_*Cl*_x_*/PC_61_BM/mod/Ag	–	−3.97	0.899	17.32	77.1	12.01	167
P3TMAHT		–	−4.31	0.899	17.10	74.1	11.28	
DMAPA‐C_60_	ITO/PEDOT:PSS/MAPbI_3‐_ *_x_*Cl*_x_*/PC_61_BM/mod/Al	–	−3.97	0.97	17.9	77	13.4	168

^a)^ Electron mobility of ETMs

^b)^ Work function of modified metal cathode.

## 4 Interlayer Functions and Principles

### 4.1 High Performance

Photovoltaics based on PSCs have achieved a certificated PCE exceeding 22%.^3^ In planar heterojunction PSCs, the pervoskite film coverage and morphology, perovskite crystal size, and charge extraction and transport property at pervoskite/HTMs or ETMs interface are all essential factors which affect the photovoltaic performance of PSCs. At early stage, Snaith and co‐workers used a dual‐source vapor deposition method to prepare film of the mixed halide perovskite CH_3_NH_3_PbI_3‐_ *_x_*Cl*_x_*.^13^ The vapor‐deposited perovskite film was extremely uniform and smooth over a range of length scales. On the contrary, the perovskite film formed by solution‐cast method was inhomogeneous and undulating. With the improved perovskite film morphology, the vapor deposition perovskite device delivered a high efficiency of 15.4% which proved the potential of planar heterojunction for achieving high efficiency. Later, Nie and co‐workers employed a solution‐based hot‐casting technique to grow continuous, pinhole‐free thin films of organometallic perovskites with millimeter‐scale crystalline grains.^94^ Owning to the reduced bulk defects and improved charge carrier mobility in large‐grain devices, this inverted planar solar cells achieved an efficiency approaching 18%.

In addition to the perovskite active layer, interlayers including ETMs and HTMs play a very important role in further improving performance of planar PSCs. Zhou et al. manipulated an effective carrier pathway across planar heterojuction PSCs through exploring the perovskite film, the ETM, and their relevant interfaces. They improved electron transport channel via doping TiO_2_ ETM with yttrium (Y‐TiO_2_) to enhance its electron concentration and modifying ITO with PEIE to reduce its WF. They also reduced carrier recombination by annealing perovskite film under controlled humidity conditions (30 ± 5% relative humidity) through an enhanced reconstruction process. These optimizations produced a high PCE of 19.3%.^44^ Large perovskite crystal grains and less grain boundaries are needed to reduce charges recombination, and interlayers under perovskite film could control their nucleation and film growth. For example, perovskite growing on nonwetting PTAA HTM showed increased nucleus spacing by suppressing heterogeneous nucleation and facilitated grain boundary migration in grain growth by imposing less drag force. The resulted film exhibited reduced grain boundary area and improved crystallinity, which reduce charge recombination to a large extent, and solar cells made via this method delivered a PCE of 18.3%.^105^ Recently, Hou and co‐workers fabricated a regular planar PSC using dopant‐free Ta‐WO*_x_*/PDCBT bilayer HTM.^89^ Tantalum doped tungsten oxide (Ta‐WO*_x_*) showed an enhanced conductivity, a distinct density of gap states, and is more effective in doping interfacial p‐type polymers as compared to WO*_x_*. The Ta‐WO*_x_*/PDCBT bilayer HTM offered a quite small interface barrier and formed quasiohmic contacts between metal electrode and HTM. The phosphonic acid C_60_‐SAMs ETM facilitated charge extraction and the growth of large crystals. Subsequently, a high PCE up to 21.2% was obtained. Moreover, some other strategies such as introducing SAMs on SnO_2_ to form chemical interactions at ETM/perovskite interface,^117^ designing new ETMs (such as HATNT ETM mentioned above) with high electron mobility and strong interfacial interaction with perovskite instead of PC_61_BM, and inserting a modifier between ETM/metal electrode were all adopted with the same principle that is to reduce trap states at perovskite/interlayers/electrode interface which act as recombination centrals, to adjust energy level alignment and to improve the charge mobility of interlayers which facilitate charge extraction and transport.^117^, ^144^, ^171^

### 4.2 Stability Issues

Along with the rapid efficiency improvement, long term operational stability of the materials and devices remains to be an obstacle for realistic implementation of PSCs. The major issues related to the stability of perovskite active layer include crystal structure stability, chemical stability under moisture, oxygen, and ultraviolet (UV) light, and interface reaction with ETMs, HTMs, or the contact electrodes. Many studies tailored perovskite stability in the molecular level, such as partial replacement of I^−^ with Br^−^ or Cl^−^ or SCN^−^, organic cation CH_3_NH_3_^+^ with Cs^+^ or HC(NH_2_)_2_^+^, or using 2D layered perovskite (PEA)_2_(MA)_2_[Pb_3_I_10_] (PEA denotes phenylethylammonium, C_6_H_5_(CH_2_)_2_NH_3_^+^)^142^, ^172^ to form moisture resistant film. Moreover, interface engineering can also help restrict chemical degradation of perovskite layer or its reaction at relevant interface.^146^, ^172^

CH_3_NH_3_PbI_3_ is extremely sensitive to the humidity in air and it is prone to decomposition by releasing CH_3_NH_3_I and leaving PbI_2_ behind, which leads to the degradation of the performance of PSCs. Based on this degradation mechanism, some studies have focus on improving the stability by introducing moisture resistance layers. Xie et al. inserted a PCBDAN interlayer between PC_61_BM and Ag cathode.^124^ The hydrophobic PCBDAN layer enhanced air stability of PSCs by effectively protecting the perovskite film from water corrosion. Li and co‐workers modified compact TiO_2_ ETM with a moisture‐resistant PCBSD:GD hybrid interlayer (cross‐linkable fullerene[6,6]‐phenyl‐C61‐butyric styryl dendron ester combined with graphdiyne) and achieved high‐efficiency PSCs with long‐term stability.^161^ Bai et al. used a water‐resistant cross‐linkable silane‐functionalized fullerene to replace hydrophilic PC_61_BM and devices based on this ETM retained nearly 90% of their original efficiencies after 30 d exposure in an ambient environment.^164^ A dopant‐free polymeric HTM (4,8‐dithien‐2‐yl‐benzo[1,2‐d;4,5‐d′]bistriazole‐alt‐benzo[1,2‐b:4,5‐b′]dithiophenes (pBBTa‐BDTs) was used in place of Spiro‐OMeTAD.^173^ The smooth, conormal pBBTa‐BDT2 coating could inhibit moisture ingress into devices and significantly enhanced device ambient stability. PEDOT:PSS, as a common used HTM in inverted devices, can encourage moisture uptake from atmosphere. Yang and co‐workers employed a hydrophobic small molecular TAPC as HTM to replace it.^68^ As a result, the TAPC‐based devices also demonstrated superior stability compared with the PEDOT:PSS‐based devices when stored in ambient circumstances.

The UV‐light soaking instability was serious in the TiO_2_‐based devices, because exposure of TiO_2_ to UV light could induce formation of oxygen vacancies, which are energetically deep trap levels responsible for severe recombination loss. A luminescent downshifting fluoropolymeric layer on the front side of PSCs could re‐emit ultraviolet light in the visible range promoted UV‐light soaking stability significantly.^174^ Reducing trap states between ETM/perovskite interface and lowering the interfacial recombination loss could also improve the UV‐light stability. For example, using a triblock fullerene derivative (PCBB‐2CN‐2C8) to passivate the TiO_2_ surface;^137^ or replacing TiO_2_ ETM with a PCBDAN:PC_61_BM bilayer or fullerene C_60_.^28^, ^151^

**Figure 9:**
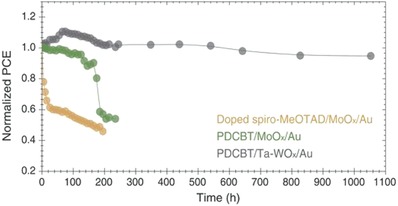
Unencapsulated device photostability tests under continuous 1 sun illumination in a home‐built chamber filled with N_2_. Reproduced with permission.^89^ Copyright 2017, The American Association for the Advancement of Science.

The decomposition of perovskite is also related to structural phase transition at elevated temperature or the interaction with the interlayers or electrode materials. Devices based on ZnO ETM always exhibited poor performance. This appearance may origin from the hydroxyl groups, organic residuals, and oxygen vacancies on the surface of the ZnO layer that can easily lead to thermal decomposition or the deprotonation of methylammonium cation (CH_3_NH_3_^+^) caused by ZnO. To address this issue, a thin layer of PC_61_BM orSnO_2_ passivation layer was inserted between the ZnO and perovskite film to prevent the perovskite decomposition at elevated temperature caused by ZnO.^141^, ^175^ The PC_61_BM ETM also facilitated the degradation of hybrid perovskite films under illumination by absorbing the decomposition products and inducing the corrosion of electrode by forming an interfacial AgI layer. To alternate the PC_61_BM ETM, a perylendiimide derivative based ETM (PDI‐EH) was reported.^114^ The compact PDI‐EH film could provide good isolation for perovskite films preventing their decomposition and leading to significantly improved device operation stability. The PEDOT:PSS HTM used in inverted PSCs is hydrophilic and acid, which not only absorbs moisture inducing perovskite decomposition but also causes the indium diffusion into the active layer and corroding the ITO electrode. Therefore, Heeger and co‐workers fabricated inverted PSCs with a pH‐neutral conjugated polyelectrolyte (CPE‐K) as HTM and improved devices stability in air.^38^ Spiro‐OMeTAD, doped with Li‐TFSI and TBP, is an effective HTM in regular structure PSCs. Besides the hydrophilic Li‐TFSI dopant, TBP is volatile and can corrode perovskite film by complexing with PbI_2_ which accelerate its degradation. Recently, Hou and co‐workers employed a conjugated polymer (PDCBT)/Ta‐WO*_x_* as HTM in n–i–p planar PSCs in place of doped Spiro‐OMeTAD.^89^ PDCBT was doped by transferring electrons to Ta‐WO*_x_* which avoided the use of dopants. The performance loss caused by the inactivation of the doped Spiro‐OMeTAD, the detrimental TBP dopants reaction with the perovskite, and reaction between perovskite and polymer was eliminated, as a result, an over 1000 h light stability was achieved, shown in **Figure** **9**. Although many advances have been made to improve the stability of PSCs, more deep understanding of the degradation mechanism under continuous operation is needed to proceed their commercialization.

### 4.3 Hysteresis‐Suppressed

Anomalous photocurrent hysteresis exists in many organometal trihalide PSCs, which may originate from several root causes, such as ion diffusion and charge blockade at interface, ferroelectric polarization, capacitive charging and interface, or bulk related charge trapping and detrapping. Previous studies showed that *J*–*V* hysteresis predominantly aroused from the presence of the perovskite absorber in the solar cells and was strongly dependent on the contact material, including p‐type and n‐type contacts, and mesoporous versus planar heterojunctions.^176^ It has been reported that the trap states on the surface and grain boundaries of the perovskite materials may be the origin of photocurrent hysteresis. Growing continuous, pinhole‐free perovskite films with millimeter‐scale crystalline grains by hot‐casting technique could suppress charge trapping and eliminate hysteresis significantly.^94^ The trap density of states (tDOS) were reduced by two orders of magnitude after PC_61_BM ETM was deposited on the perovskite film and permeated into the perovskite layers along the grain boundaries during thermal annealing.^177^ This showed the passivation effect of PC_61_BM could reduce the *J*–*V* hysteresis. Later, Gerbaldi and co‐workers pointed the dynamic process of the interfacial charge governs the current–voltage hysteresis.^178^ The accumulation of mobile ions caused space charge at interfaces and ion redistribution process was slow when the bias was changed. The interfacial charges influenced band bending and thus the photocurrent of the solar cells. The thickness of the carrier‐selective layer (PC_61_BM) significantly affected the interfacial charge density and they fabricated a hysteresis‐free device by controlling the thickness of PC_61_BM. In addition to the passivation effect of PC_61_BM, this hysteresis‐free property of inverted planar PSCs can also attribute to the balanced electron flux and hole flux, because the electron extraction at perovskite/PC_61_BM interface is improved than that at perovskite/TiO_2_ interface.^83^ Therefore, much exploring has been proceeded to adjust ETM/perovskite interface by using fullerene or other organic ETMs. In regular devices, Snaith and co‐workers replaced the commonly employed TiO_2_ compact layer with fullerene C_60_ in a regular architecture.^28^ The steady state photoluminescence (PL) and time‐resolved PL measurements both proved the enhanced electron transfer efficiency at perovskite/C_60_ than that at perovskite/TiO_2_ interface which interpreted the negligible hysteresis in the C_60_‐based devices. Kim et al. described a bilayered ETM combining fulleropyrrolidinium iodide (FPI)‐polyethyleneimine (PEIE) and PC_61_BM in regular PSCs.^149^ The combination of WF modification effect of FPI‐PEIE and the charge extraction of PC_61_BM led to high efficiency solar cells with insignificant hysteresis. Wang and co‐workers used a fullerene derivative CPTA as ETM in regular PSCs.^152^ The electron mobility of CPTA is twice as high as that of PC_61_BM, which is widely used as an efficient ETM in photovoltaic devices. This property can lower charge accumulation at the interface and balance carrier fluxes between CPTA and doped Spiro‐OMeTAD and suppressing the hysteresis effect to a great extent. Except for fullerene‐based materials, Yang and co‐workers used solid‐state ionic‐liquids (1‐benzyl‐3‐methylimidazolium chloride) as ETM to replace TiO_2_ in regular devices.^156^ This ETM could reduce the electron trap‐state density of the perovskite absorber and balance carrier fluxes due to its high electron mobility, as a result, the hysteresis was effectively prevented. Moreover, modifying the surface of TiO_2_ or WO*_x_* ETMs with fullerene or fullerene SAM molecules is also a useful way to solve the serious hysteresis issue. For instance, Wojciechowski et al. modified TiO_2_ ETM with a self‐assembled fullerene monolayer (C_60_‐SAM) and Hou and co‐workers modified WO*_x_* ETM with mixed fullerene functionalized SAM.^133^, ^143^ All these works relieved hysteresis by taking advantages of the improved electron transfer efficiency and the reduced trap states density after insertion of SAMs at ETM and perovskite interface.

### 4.4 Large‐Area Devices

PSCs are a promising low‐cost and highly efficient photovoltaic technology. However, most of the reported high efficiencies were obtained on a small working area of about 0.1 cm^2^ in the laboratory research. To advance the application of PSCs, solar cells with large area and uniform perovskite films as well as the interlayers are pressing to be fabricated. Some strategies such as vacuum flash‐assisted solution processing method, soft‐cover deposition method, blade‐coating method, and solvent‐free and vacuum‐free approach followed by a pressure application step are applied to form high‐quality, large‐area perovskite film.^179^, ^180^, ^181^, ^182^ Meanwhile, interface engineering for preparation of large‐area PSCs has also been developed, including forming interlayers which do not need delicate control of their thickness, with perfect wettability to form full‐coverage and large‐grain perovskite film or are compatible to printing film‐forming method.^104^, ^150^, ^181^ Only when both perovskite and interlayers were formed at large area and high quality, the high efficiency large‐area devices could be fabricated.

### 4.5 Flexible Devices

Most academic and industrial researches have focused on the progress of PSCs on rigid glass substrates. Nevertheless, due to the simple processing (either vaporation or solution) and low forming temperature (<150 °C) of perovskite absorber, flexible PSCs are growing rapidly. The widely used flexible substrates are polyethylene terephthalate (PET) film, polyethylene naphthalate (PEN) film, and metal substrates.^183^ In n–i–p flexible PSCs, fullerene based ETMs such as C_60_,^184^ PC_61_BM,^148^ and CPTA,^152^ and ionic liquid^156^ with relatively low processing temperature are more suitable than the metal oxide semiconductors (such as TiO_2_,^185^ ZnO,^186^ and Zn_2_SnO_4_ nanoparticles^82^) for fabrication of flexible devices. In p–i–n flexible PSCs, the HTM layer is typically deposited by spin coating PEDOT:PSS, while PC_61_BM is mainly used as the ETM.^32^, ^187^ Both of them can effectively extract charges and are compatible with flexible devices processing. Although the efficiency and bend resistance are improved significantly, the stability issue and roll‐to‐roll processing technologies of flexible devices need to be further studied for their industrialization.

## 5 Conclusion and Perspectives

Planar PSCs have induced growing interest in recent years since the advantages of high performance, low cost, and ease of fabrication via solution processes. Efforts to optimize the interlayers include the following four espects: 1) better WF alignment with perovskite layer by adjusting the side chains to impove charges transfer; 2) high charge mobility with good conjugated planarity, large conjugated units, or fullerenes for effective charge extraction and transport; 3) proper interfacial property to optimize the perovskite film growth; 4) new interlayers to improve device stability. Organic interlayers with the great molecular structure tunability offer a bright future for the optimization of planar PSCs.

Although PCE of the best planar PSCs is relatively low compared to the mesoscopic devices, the performance of planar PSCs has been improved through continuously modifying the perovskite/interlayer/electrode interface. The modification principle of organic interlayers is based essentially on the adhesion force of underlying layers (e.g., transparent conductive oxides or perovskite crystals) and the ability to adjust the crystallinity of the perovskite. On the transparent conductive layer, the groups capable of reacting with a hydroxyl group are usually the suitable choice. An organic interlayer on the perovskite materials typically exhibts electron donating groups to passivate the active layer.^188^ Device stability can also be improved by rigorous molecular structure device. Hydrophobic small molecules or cross‐linked polymers, dopant‐free and structure stable materials, or nonreactive materials to perovskite are all potential candidates. A trend in this field should be focused on: 1) allowing the organic interlayers to be more versatile, such as the implantation of the nucleation seeds;^189^ 2) mixing active layer with the functional organic molecules to increase the reaction area between them; 3) designing protective interlayers to prevent perovskite degradation.

## Conflict of Interest

The authors declare no conflict of interest.
